# Supplementary material for: Highly efficient multi-resonance thermally activated delayed fluorescence material toward a BT.2020 deep-blue emitter
Source: Nat Commun. 2024 Apr 2;15:2361. doi: 10.1038/s41467-024-46619-8 (PMC10987657; doi:10.1038/s41467-024-46619-8)
Supplement: Supplementary file 1 — Supplementary Information [file 41467_2024_46619_MOESM1_ESM.pdf]

Supplementary Figures

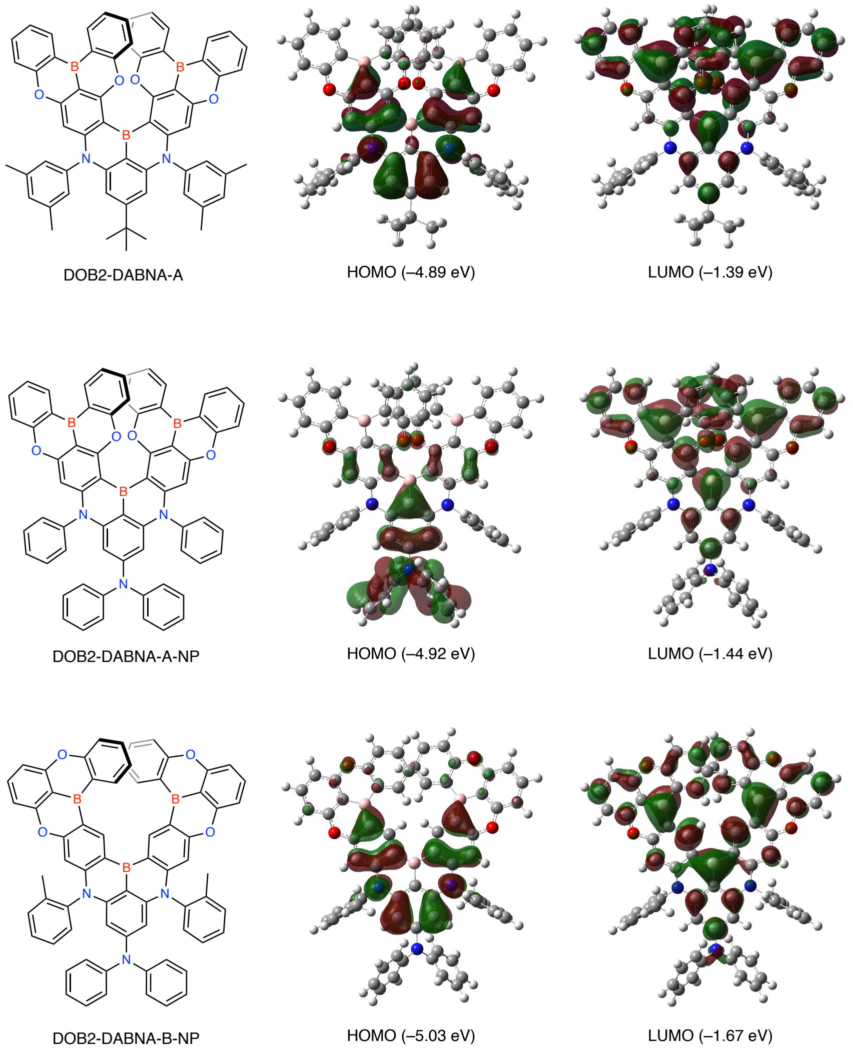

Supplementary Figure 1: Kohn–Sham frontier orbitals of DOB2-DABNA-A, DOB2-DABNA-A-NP, and DOB2-DABNA-B-NP. They were calculated at the B3LYP/6-31G(d) level of theory (isovalue = 0.02).

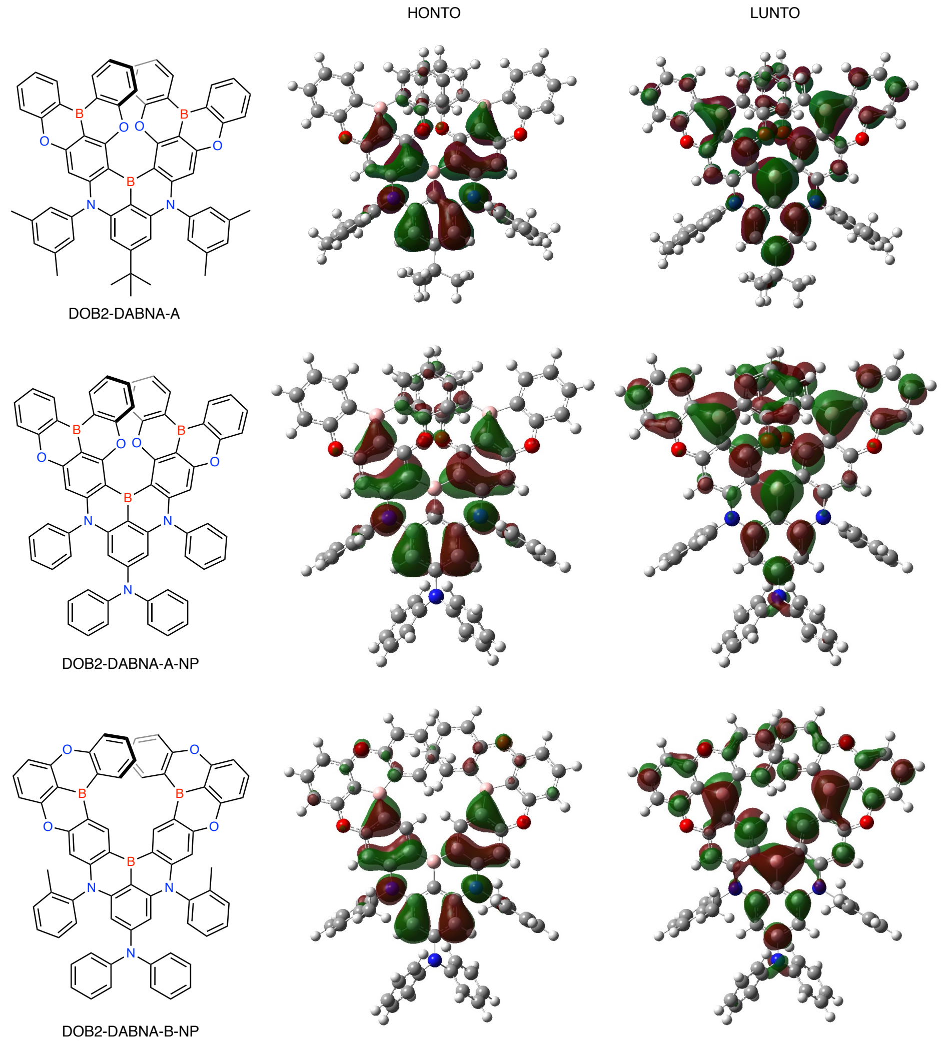

Supplementary Figure 2: Natural transition orbitals in the S_0–S_1 transition of DOB2-DABNA-A, DOB2-DABNA-A-NP, and DOB2-DABNA-B-NP. They were calculated at the B3LYP/6-31G(d) level of theory (isovalue = 0.02).

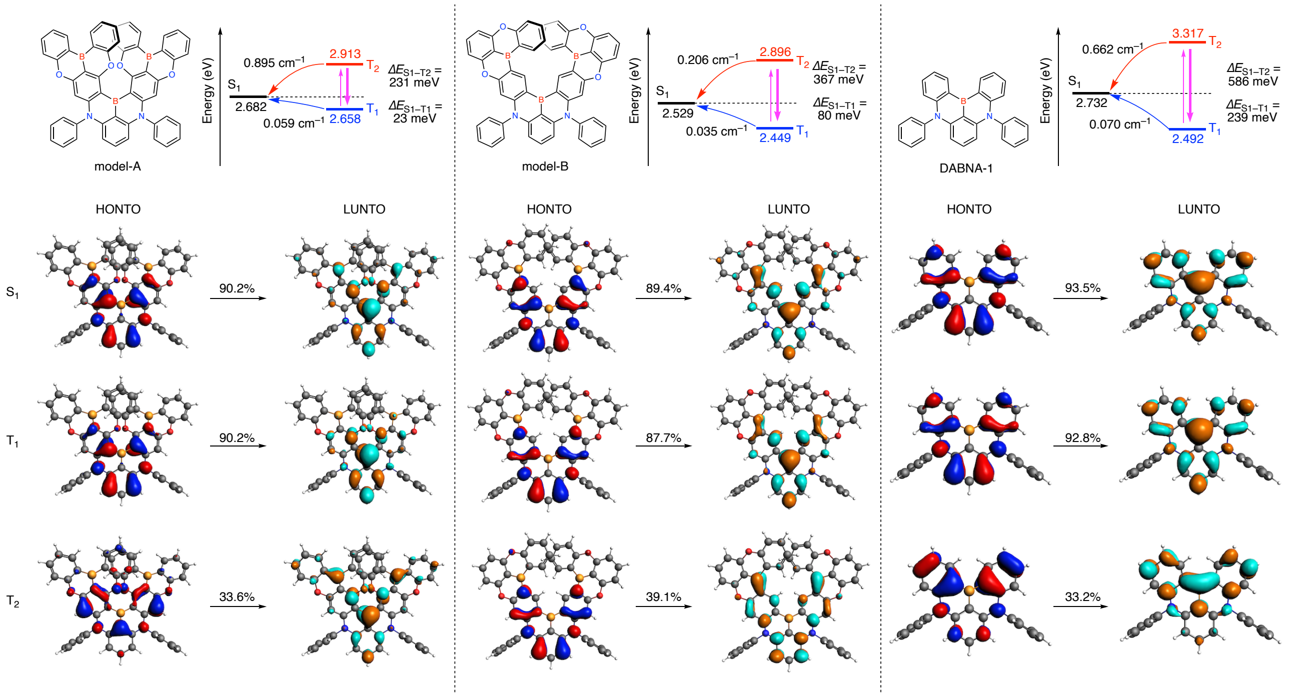

Supplementary Figure 3: Energy-level diagrams and related natural transition orbitals (NTOs) for the singlet and triplet excited states of model-A, model-B, and DABNA-1 with S_1 geometry. Transition energies for S_1, T_1, and T_2 were calculated at the TDA-B2PLYP(cx=0.40, cc=0.23)/cc-PVDZ//M062X/6-31G(d) levels of theory. NTOs and SOC matrix elements were calculated at the M062X/TZP//M062X/6-31G(d) level of theory.

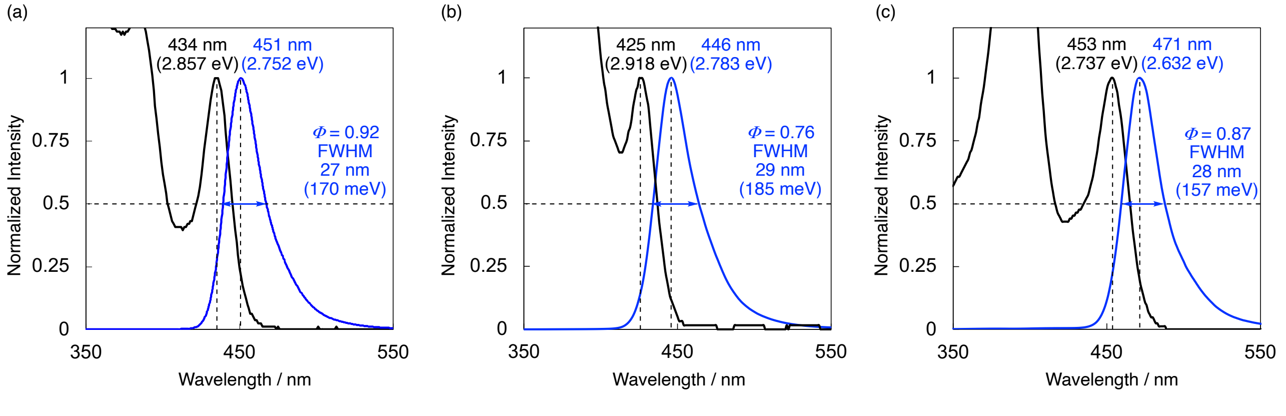

Supplementary Figure 4: **Normalized absorption (black) and fluorescence (blue, excited at 340 nm) spectra**. The spectra of (a) DOB2-DABNA-A, (b) DOB2-DABNA-A-NP, and (c) DOB2-DABNA-B-NP in 1 wt-% doped poly(methyl methacrylate) (PMMA) film are shown with absorption/emission maxima (nm, eV), absolute fluorescence quantum yield (*Φ*), and full width at half maximum (nm, meV).

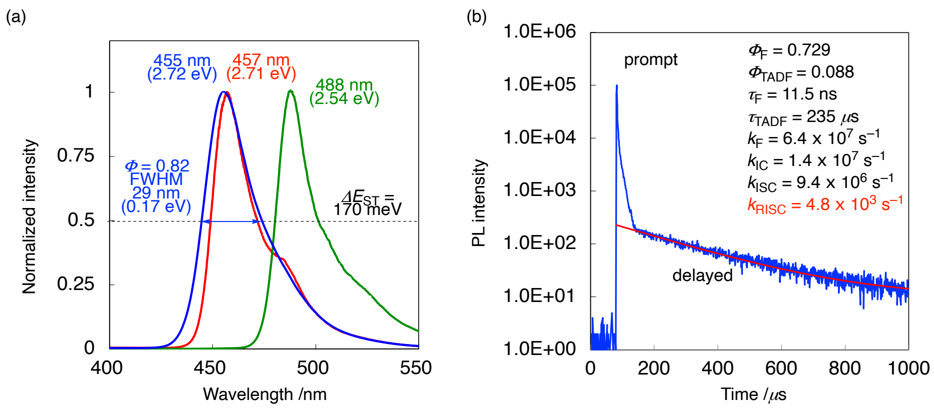

Supplementary Figure 5: Photophysical properties of DABNA-1 in 1 wt%-doped PMMA films. (a) Photoluminescence spectra at 300 K (blue) and 77 K with (green) and without (red) a delay time of 25 ms. (b) Transient photoluminescence (PL) decay curves at 300 K and their relevant parameters. The red curves represent the single exponential fitting data (background = 2).

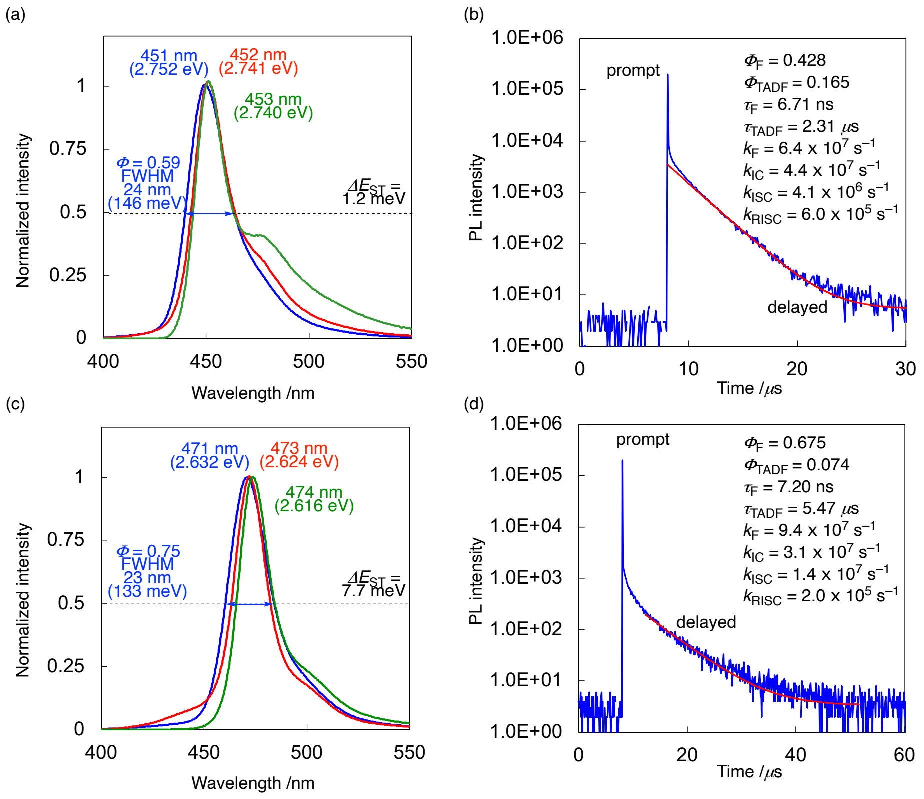

Supplementary Figure 6: Photophysical properties of DOB2-DABNA-A (a,b) and DOB2-DABNA-B-NP (c,d) in 1 wt%-doped DOBNA-Tol films. (a,c) Photoluminescence spectra at 300 K (blue) and 77 K with (green) and without (red) a delay time of 25 ms. (b,d) Transient photoluminescence (PL) decay curves at 300 K and their relevant parameters. The red curves represent the single exponential fitting data (background = 3.32–5.32).

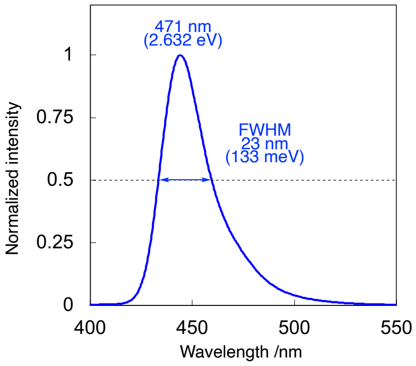

Supplementary Figure 7: Fluorescence spectrum of DOB2-DABNA-A in 0.1 wt%-doped PMMA film at 300 K. The spectrum is shown with emission maxima (nm, eV) and full width at half maximum (nm, meV).

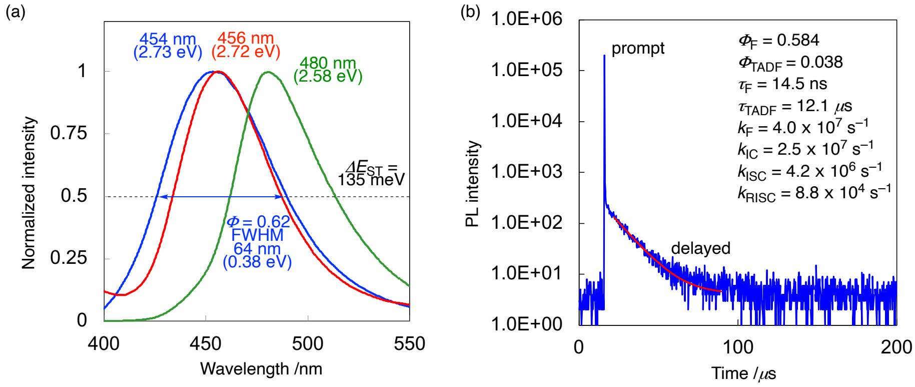

Supplementary Figure 8: Photophysical properties of a neat film of DOBNA-Tol. (a) Photoluminescence spectra at 300 K (blue) and 77 K with (green) and without (red) a delay time of 25 ms. (b) Transient photoluminescence (PL) decay curves at 300 K and their relevant parameters. The red curves represent the single exponential fitting data (background = 4).

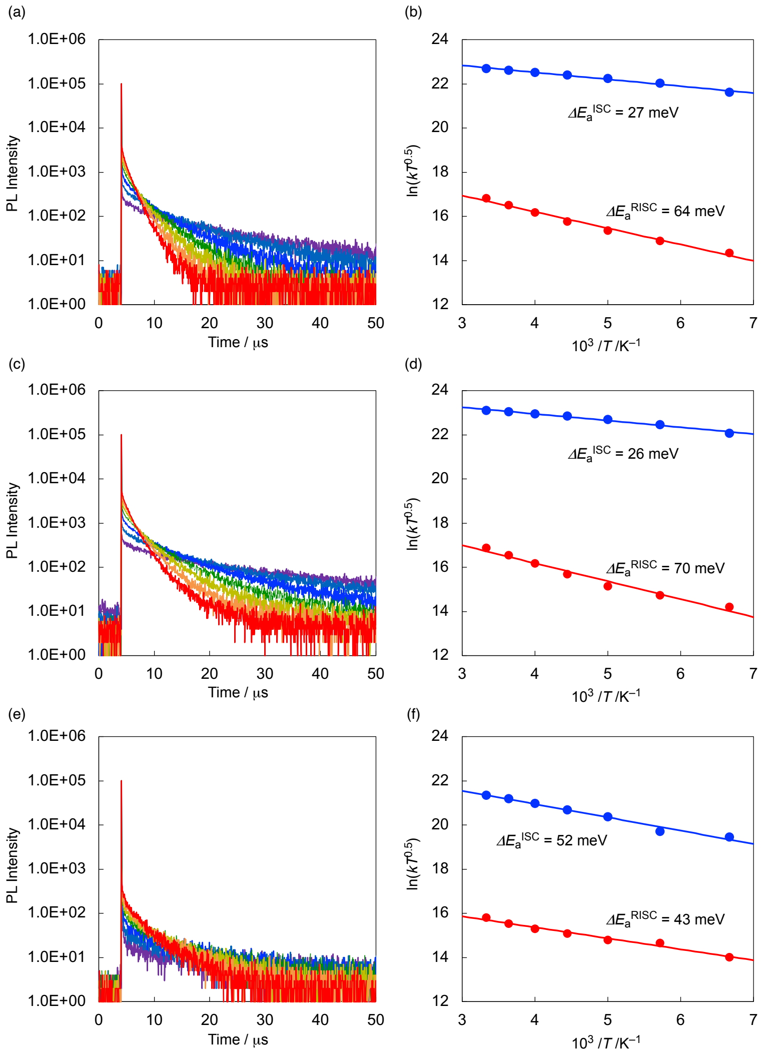

Supplementary Figure 9: Photophysical properties of (a,b) DOB2-DABNA-A, (c,d) DOB2-DABNA-A-NP, and (e,f) DOB2-DABNA-B-NP in 1 wt-% doped PMMA films. (a,c,e) Transient decay spectra at 150 (purple), 175 (indigo), 200 (blue), 225 (green), 250 (yellow), 275 K (orange), and 300 K (red). (b,d,f) Arrhenius plots of $k_{\mathrm{RISC}}T^{0.5}$ (red) and $k_{\mathrm{ISC}}T^{0.5}$ (blue) vs. $1/T$.

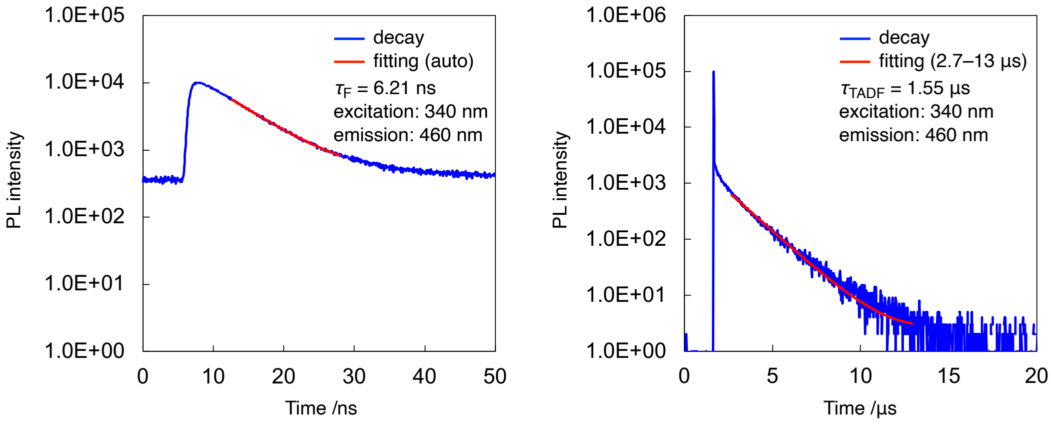

Supplementary Figure 10: Transient decay spectra of DOB2-DABNA-A in 1 wt-% doped PMMA film. A red curve is single exponential fitting data.

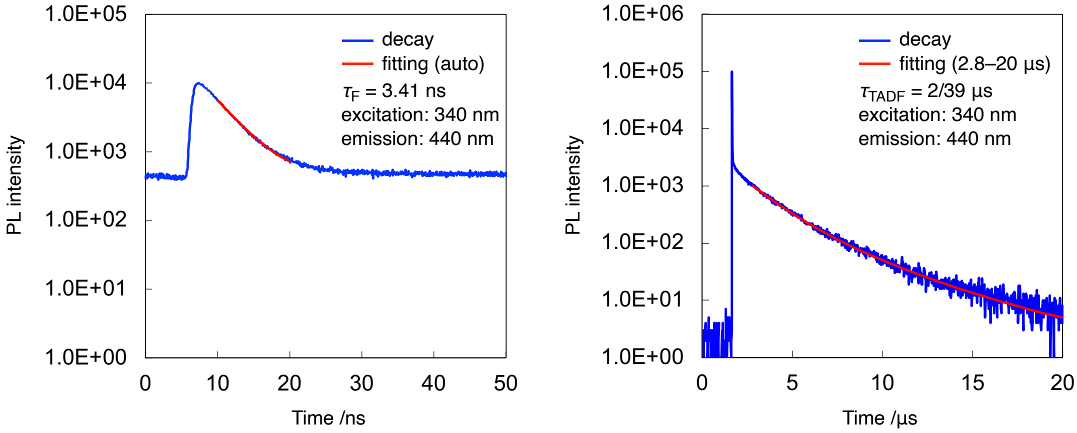

Supplementary Figure 11: Transient decay spectra of DOB2-DABNA-A-NP in 1 wt-% doped PMMA film. A red curve is single exponential fitting data.

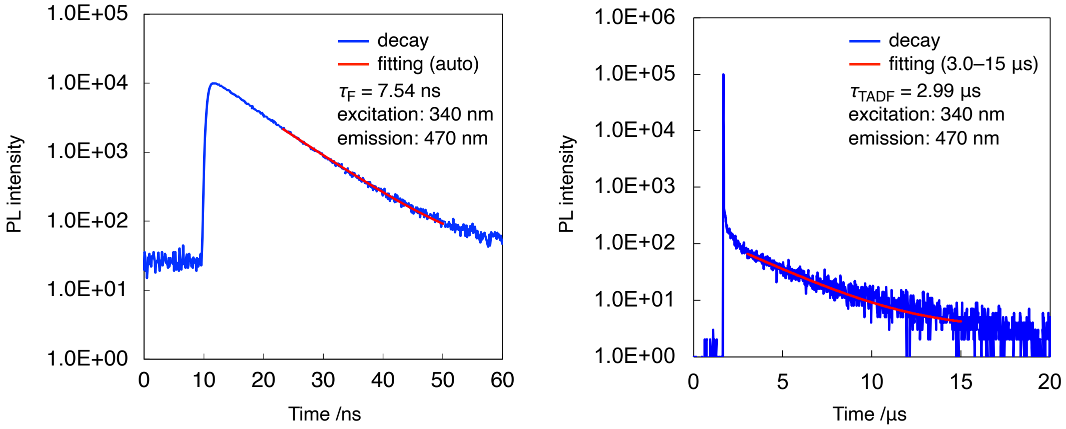

Supplementary Figure 12: Transient decay spectra of DOB2-DABNA-B-NP in 1 wt-% doped PMMA film. A red curve is single exponential fitting data.

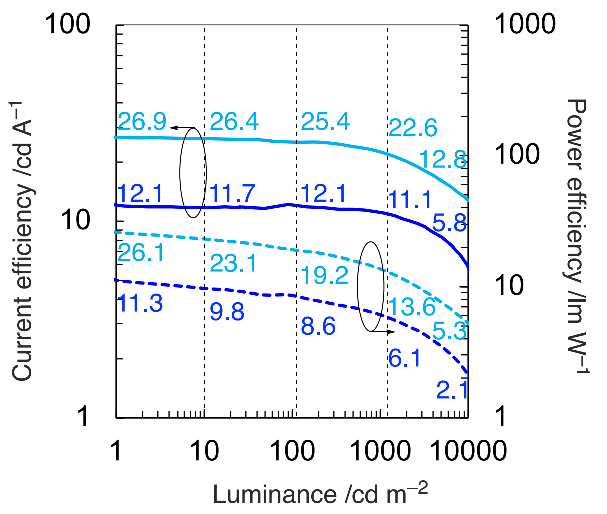

Supplementary Figure 13: Current efficiency (solid) and power efficiency (dashed) versus luminance of OLED device. Blue lines represent the device fabricated with DOB2-DABNA-A, and light blue ones represent that fabricated with DOB2-DABNA-B-NP.

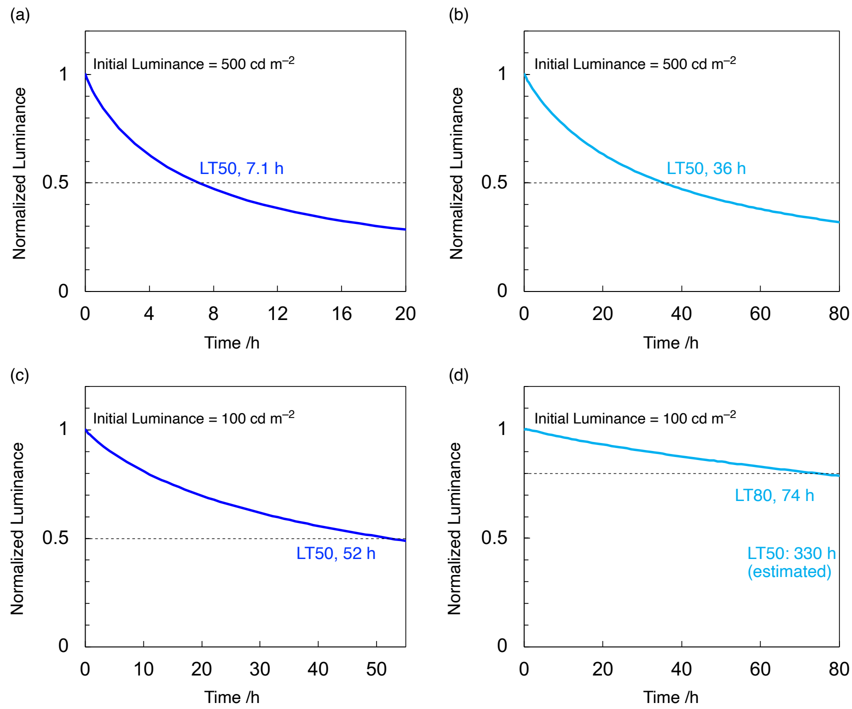

Supplementary Figure 14: Normalized luminance versus time characteristics of OLED device employing DOB2-DABNA-A (blue) and DOB2-DABNA-B-NP (light blue) with initial luminance at (a,b) 500 and (c,d) 100 cd m^{-2}. The LT50 value of DOB2-DABNA-B-NP was estimated by the equation: [LT50@100 cd m^{-2}] = [LT50@500 cd m^{-2}] × (500/100)^{n}. n was estimated to be 1.39 by fitting the data at 500 cd m^{-2} (b) to that at 100 cd m^{-2} (d).

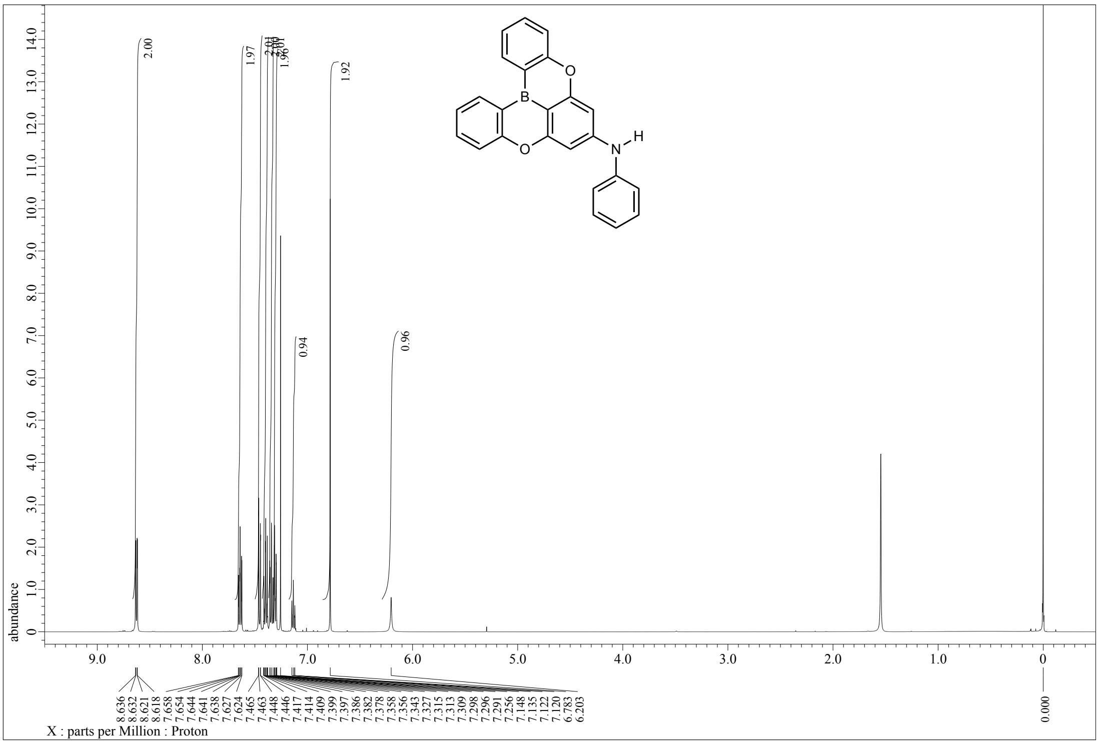

Supplementary Figure 15: **NMR data.** ^{1}H NMR spectrum of DOBNA-NHPh in $CDCl_3$ at 25 °C.

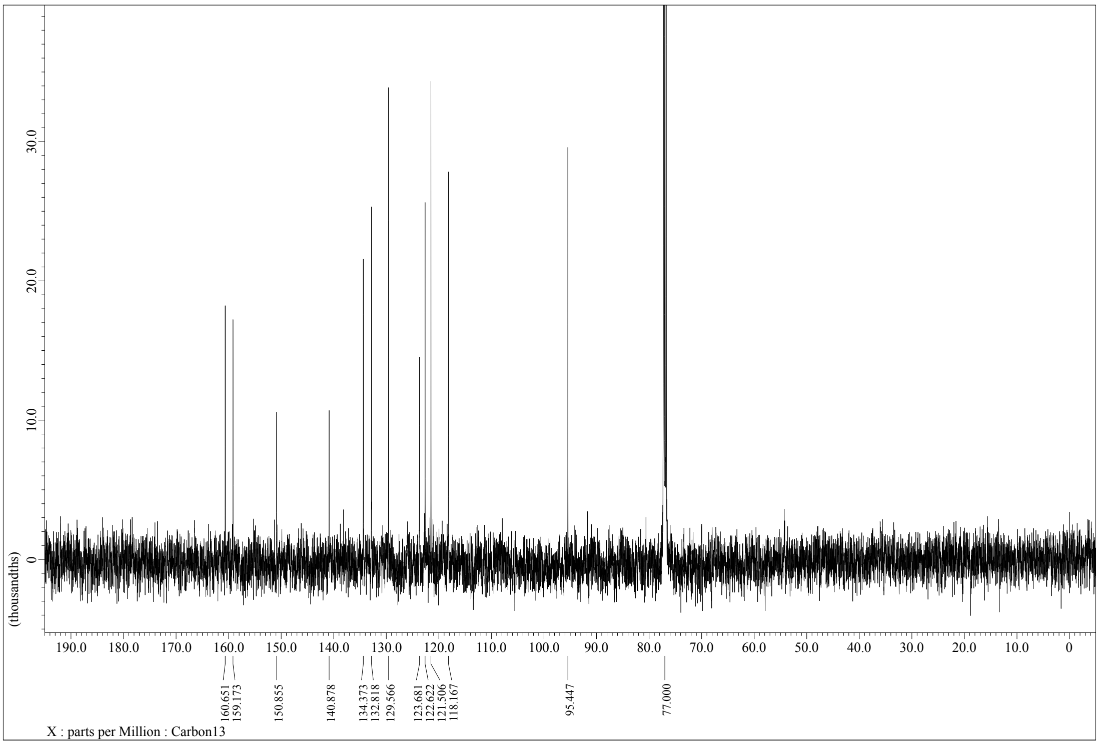

Supplementary Figure 16: **NMR data.** ^{13}C NMR spectrum of DOBNA-NHPh in $CDCl_3$ at 25 °C.

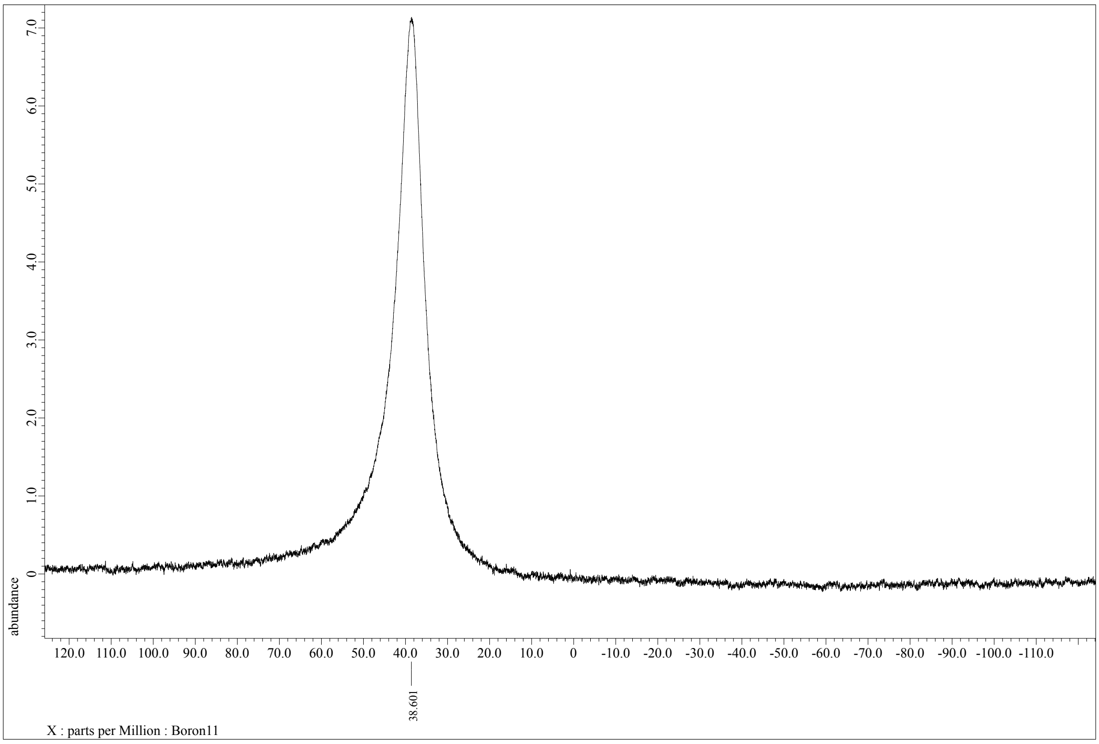

Supplementary Figure 17: **NMR data.** ^{11}B NMR spectrum of DOBNA-NHPh in $CDCl_3$ at 25 °C.

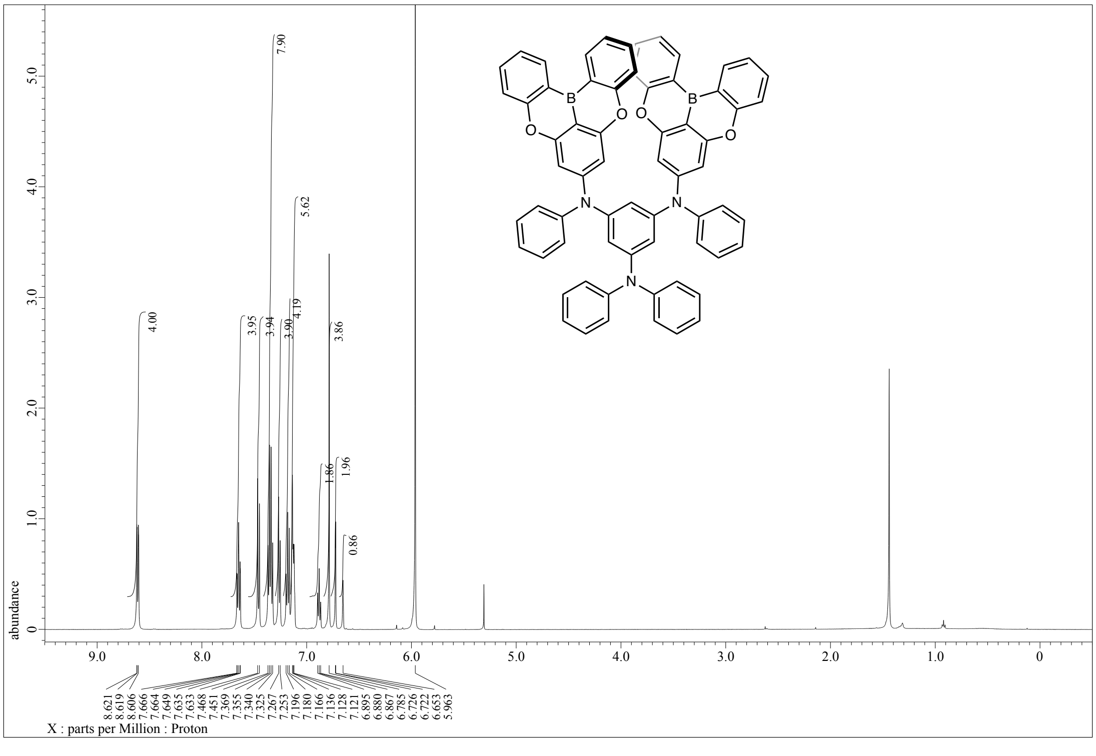

Supplementary Figure 18: **NMR data.** ^{1}H NMR spectrum of **1** in tetrachloroethane–d_2 at 100 °C.

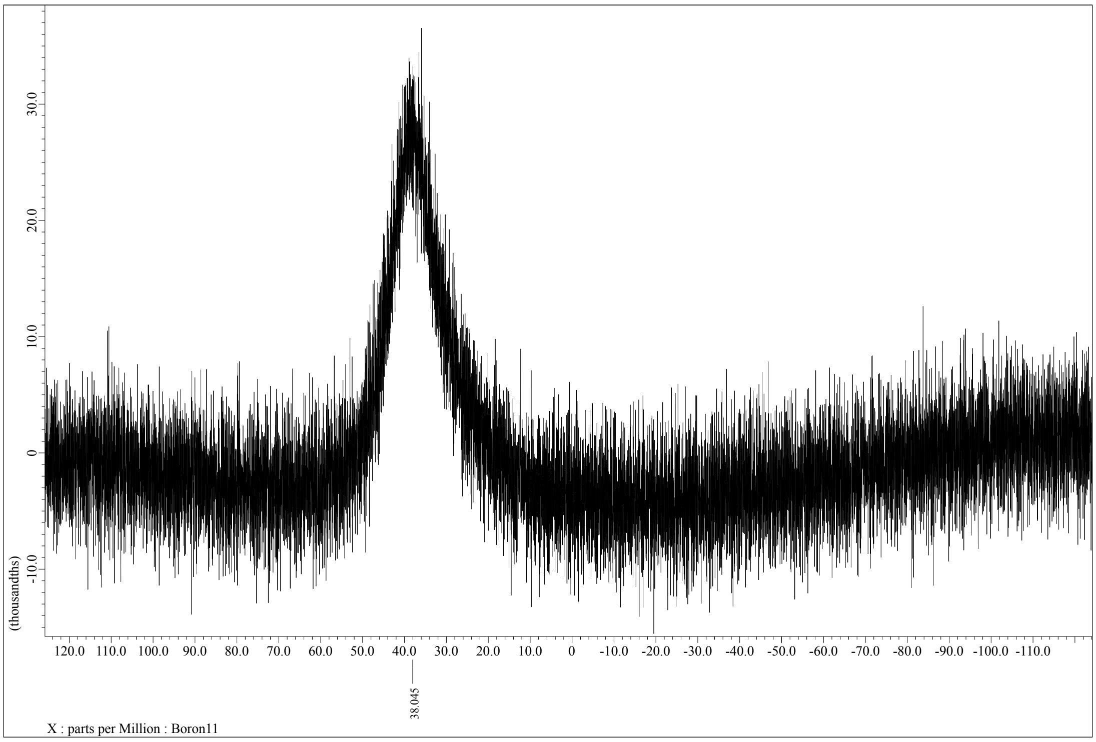

Supplementary Figure 19: **NMR data.** ^{11}B NMR spectrum of **1** in tetrachloroethane–d_2 at 100 °C.

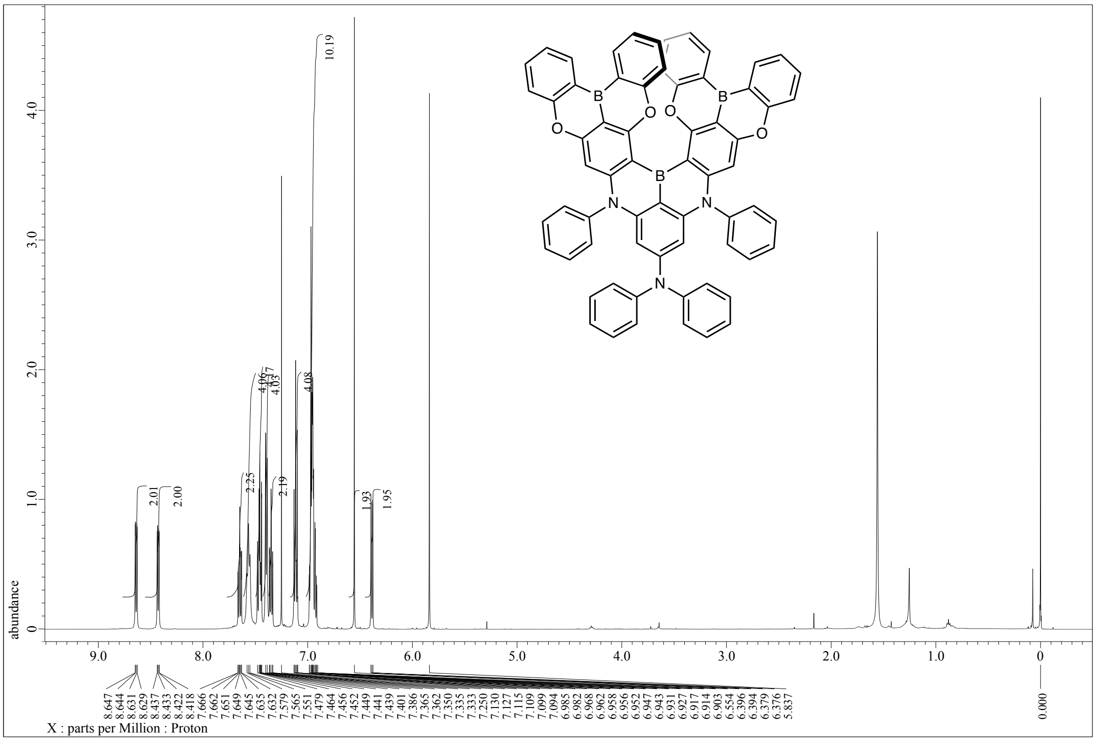

Supplementary Figure 20: **NMR data.** ^{1}H NMR spectrum of DOB2-DABNA-A-NP in $CDCl_3$ at 25 °C.

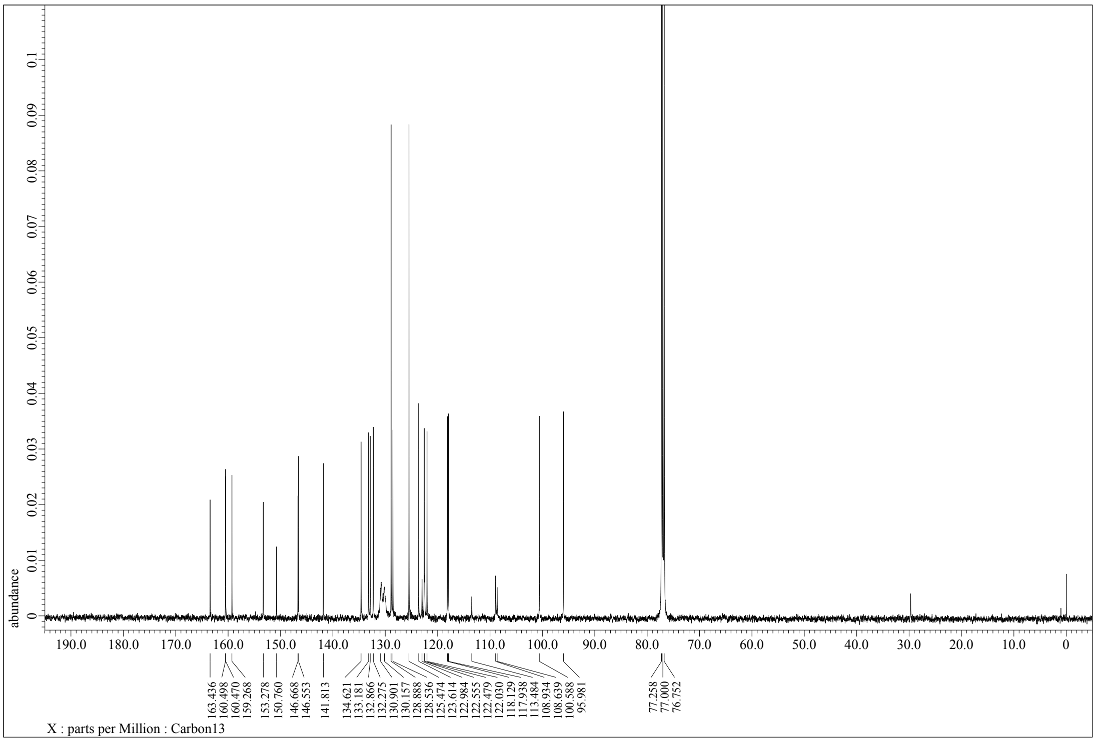

Supplementary Figure 21: **NMR data.** ^{13}C NMR spectrum of DOB2-DABNA-A-NP in $CDCl_3$ at 25 °C.

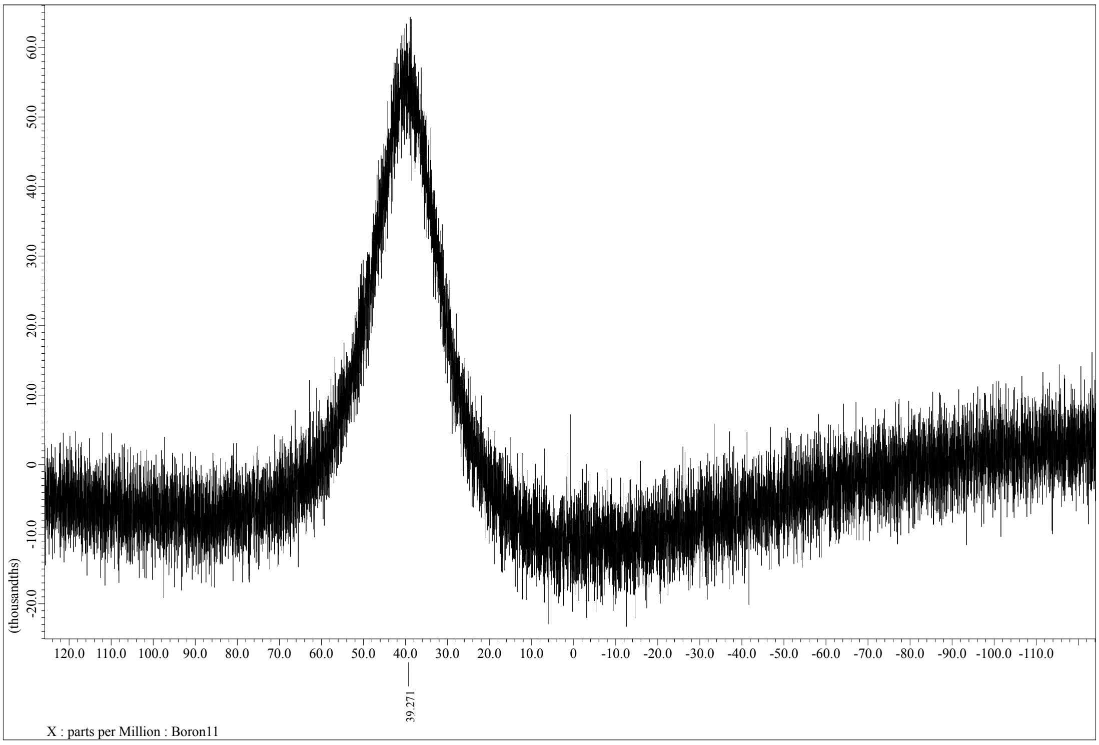

Supplementary Figure 22: **NMR data.** ^{11}B NMR spectrum of DOB2-DABNA-A-NP in $CDCl_3$ at 25 °C.

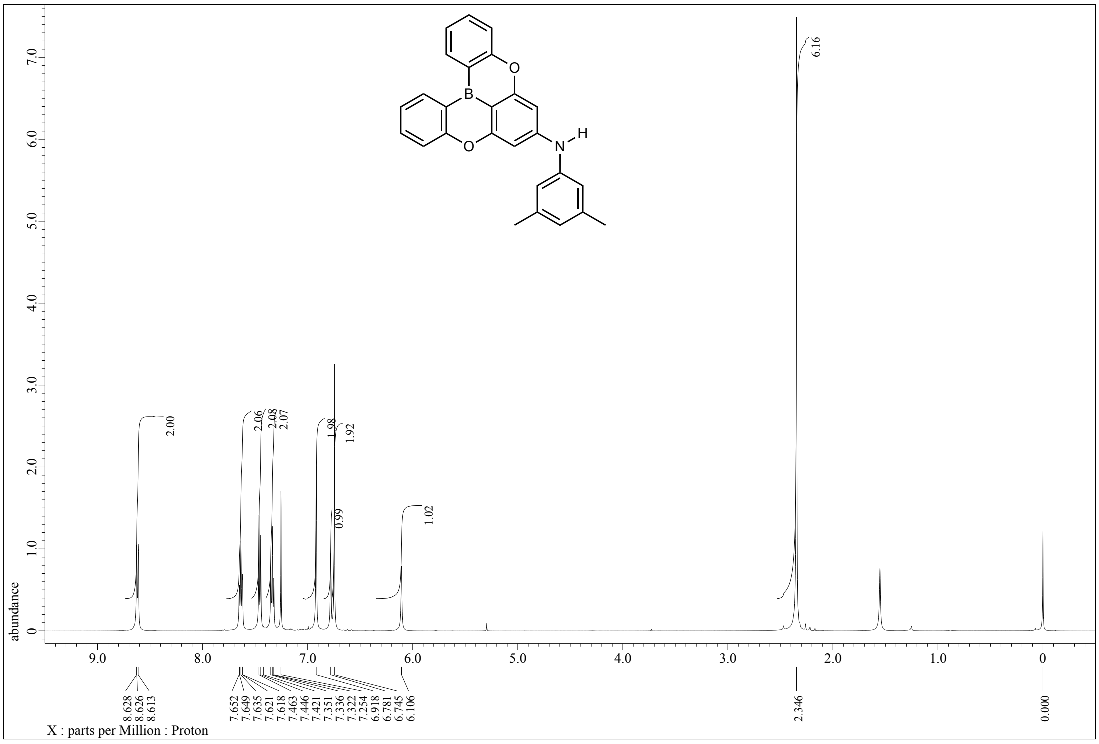

Supplementary Figure 23: **NMR data.** ^{1}H NMR spectrum of DOBNA-NHXyl in $CDCl_3$ at 25 °C.

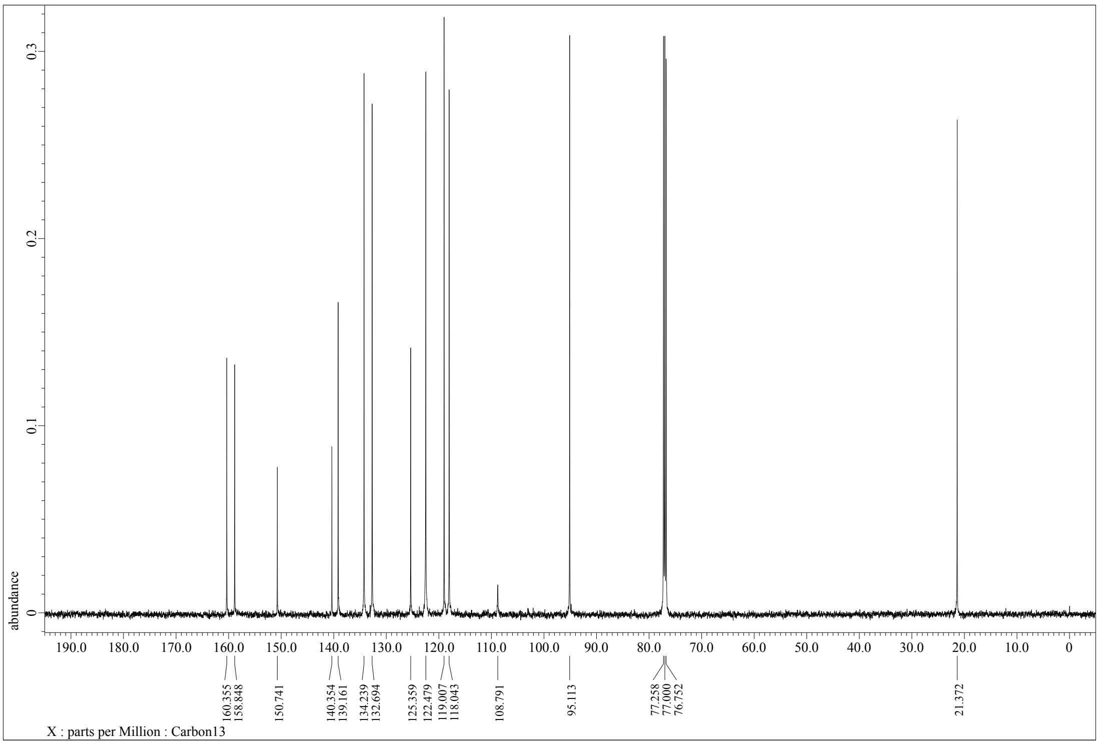

Supplementary Figure 24: **NMR data.** ^{13}C NMR spectrum of DOBNA-NHXyl in $CDCl_3$ at 25 °C.

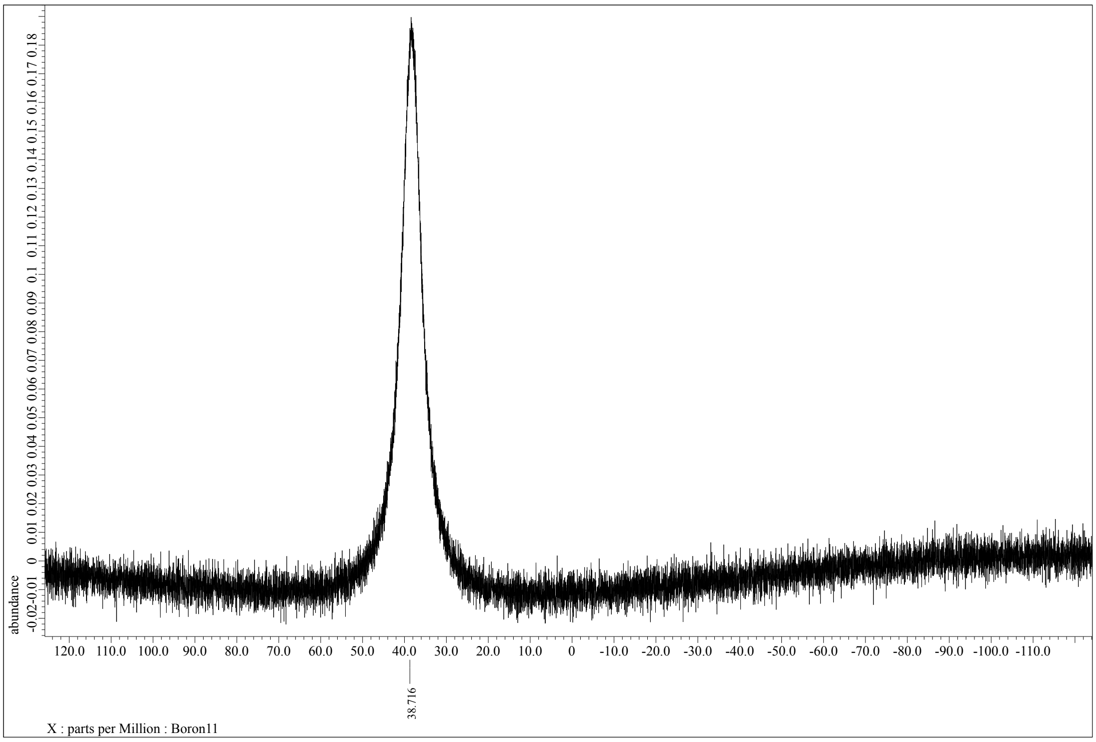

Supplementary Figure 25: **NMR data.** ^{11}B NMR spectrum of DOBNA-NHXyl in $CDCl_3$ at 25 °C.

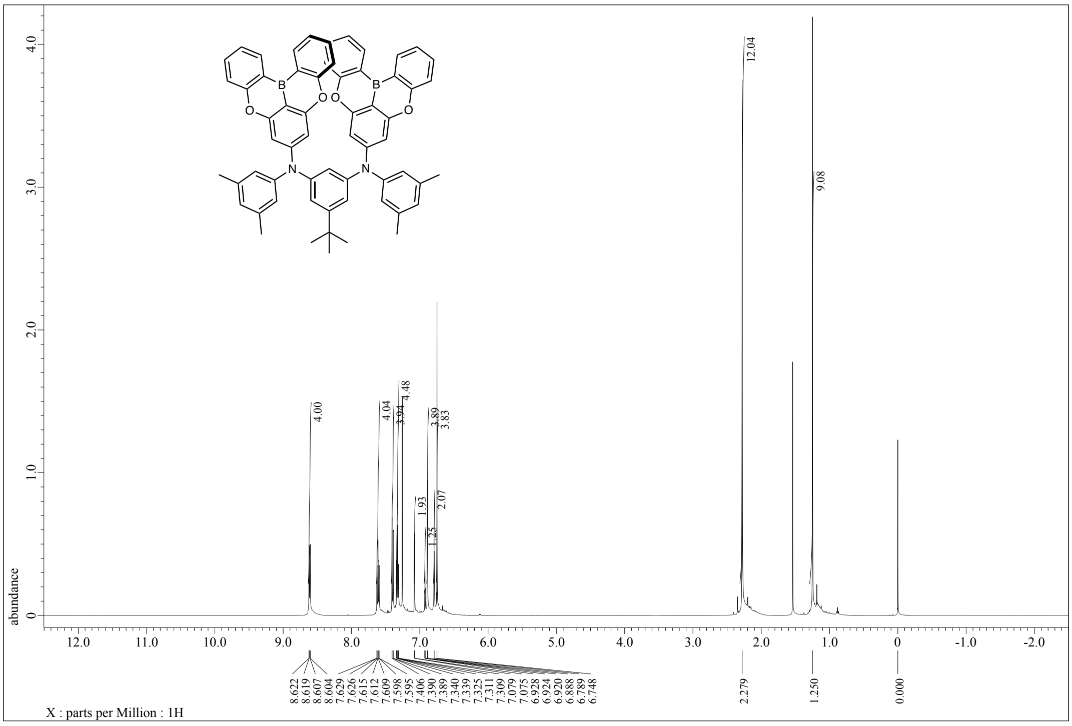

Supplementary Figure 26: **NMR data.** ^{1}H NMR spectrum of **2** in $CDCl_3$ at 25 °C.

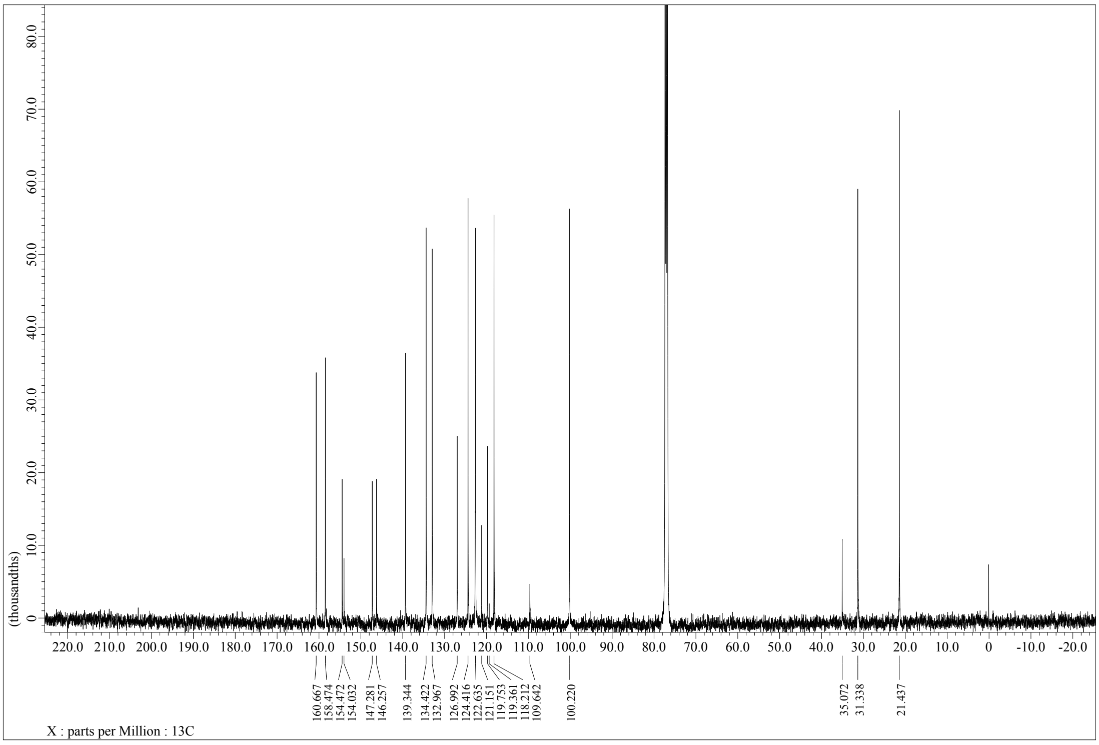

Supplementary Figure 27: **NMR data.** ^{13}C NMR spectrum of **2** in $CDCl_3$ at 25 °C.

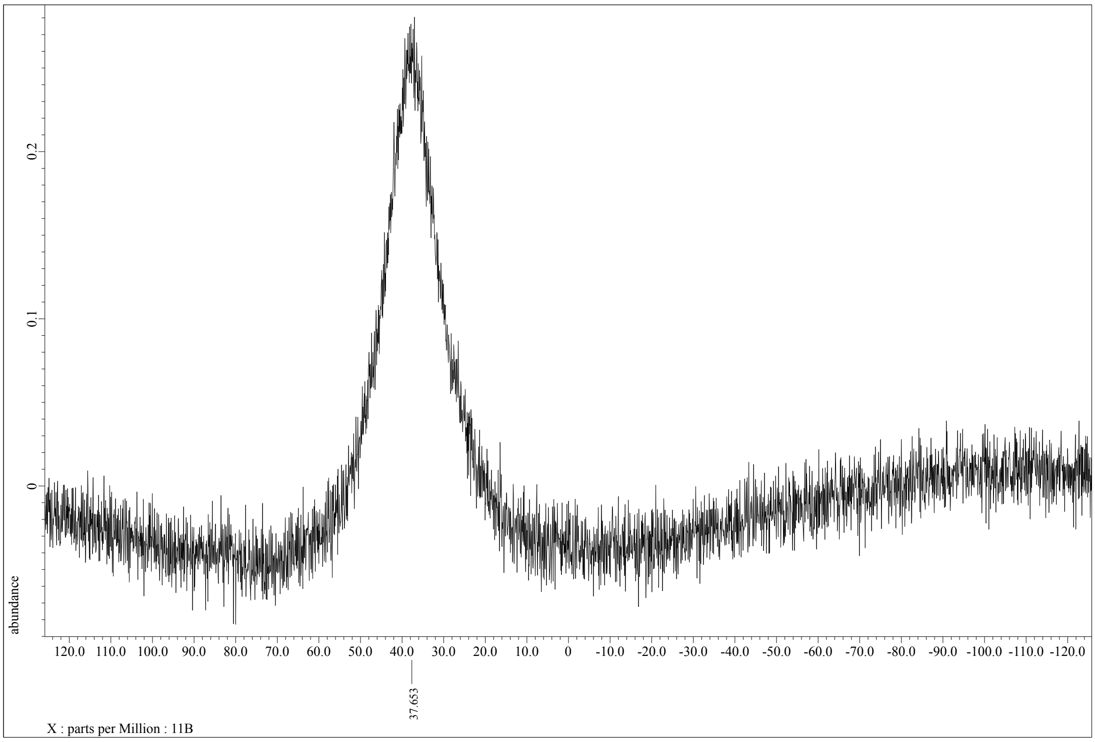

Supplementary Figure 28: **NMR data.** ^{11}B NMR spectrum of **2** in $CDCl_3$ at 25 °C.

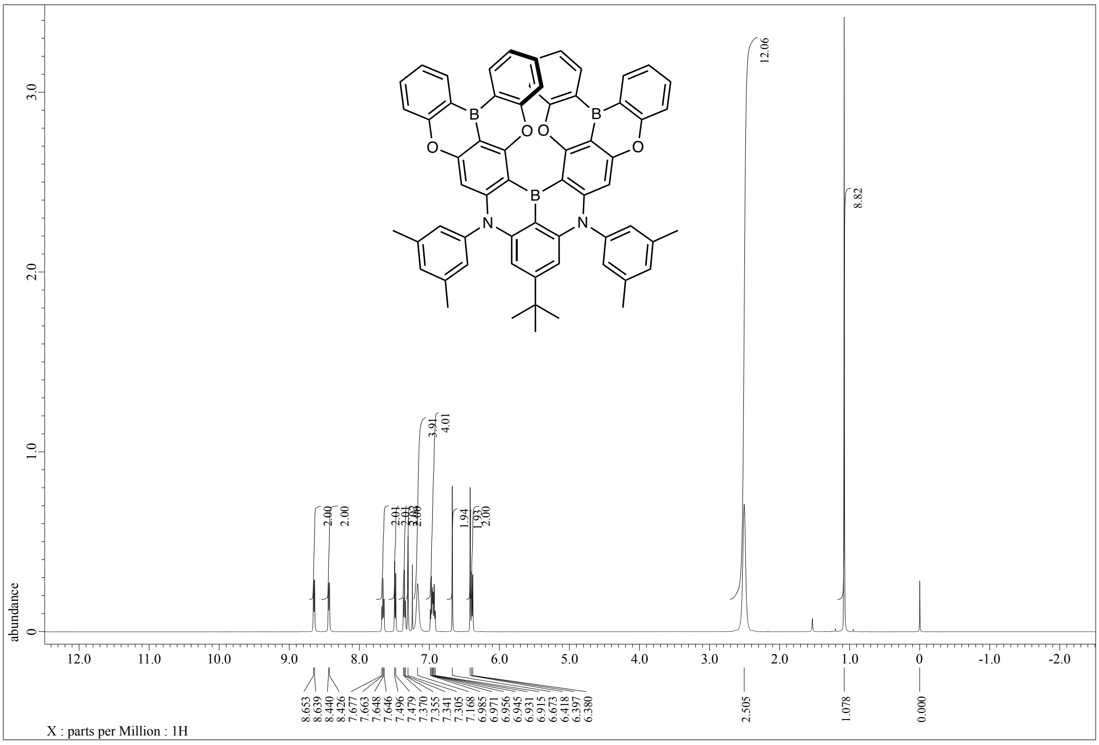

Supplementary Figure 29: **NMR data.** ^{1}H NMR spectrum of DOB2-DABNA-A in $CDCl_3$ at 25 °C.

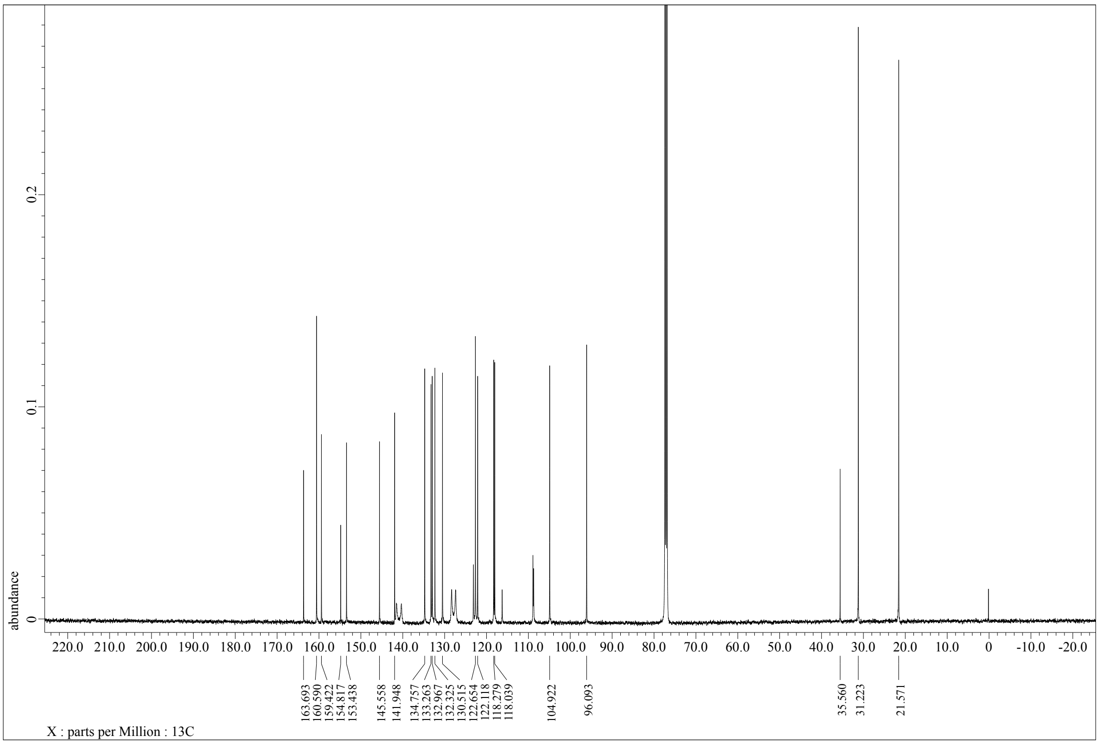

Supplementary Figure 30: **NMR data.** ^{13}C NMR spectrum of DOB2-DABNA-A in $CDCl_3$ at 25 °C.

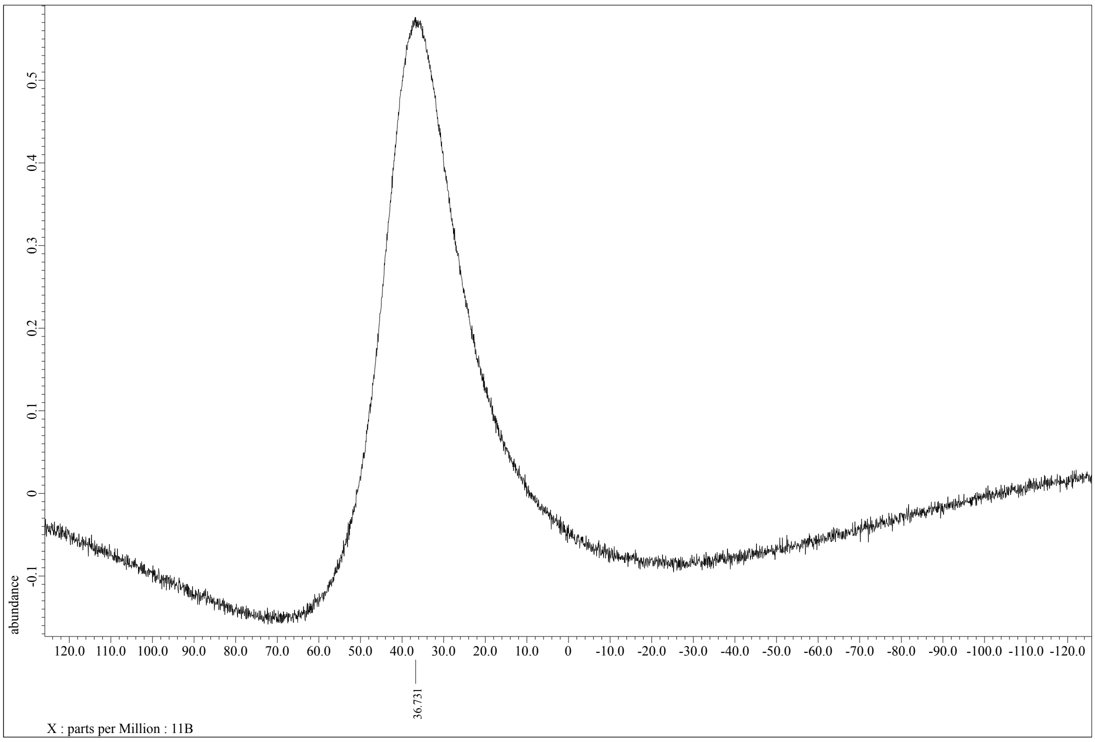

Supplementary Figure 31: **NMR data.** ^{11}B NMR spectrum of DOB2-DABNA-A in $CDCl_3$ at 25 °C.

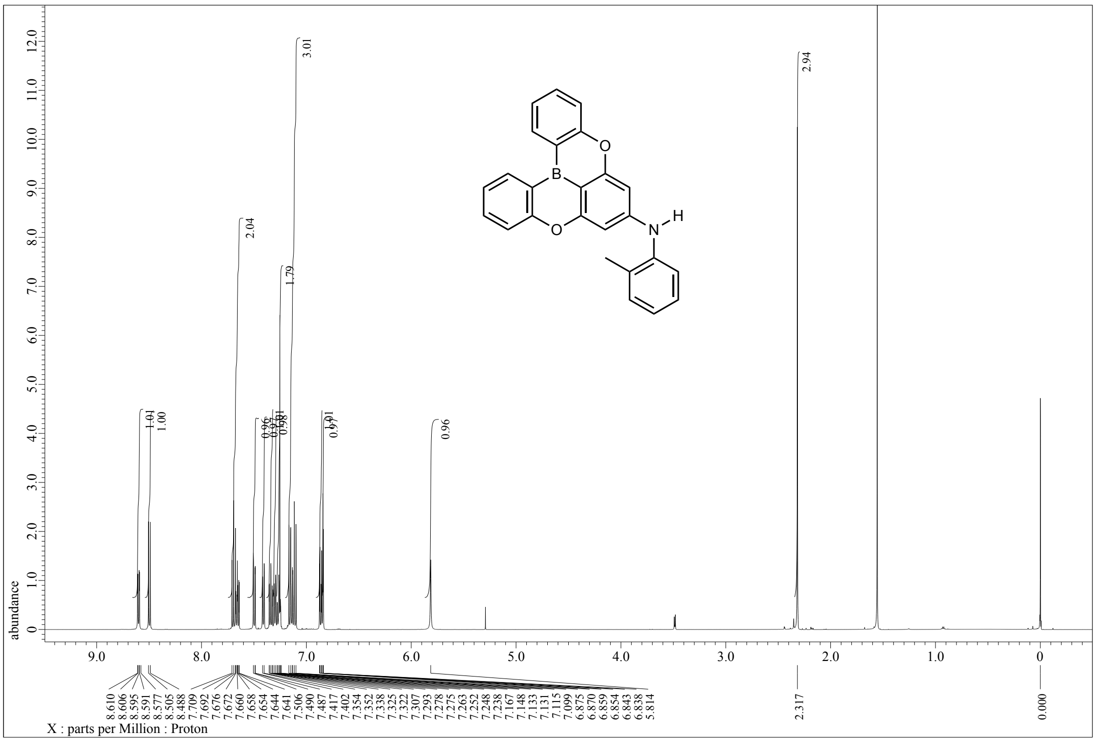

Supplementary Figure 32: **NMR data.** ^{1}H NMR spectrum of DOBNA-NH*o*Tol in $CDCl_3$ at 25 °C.

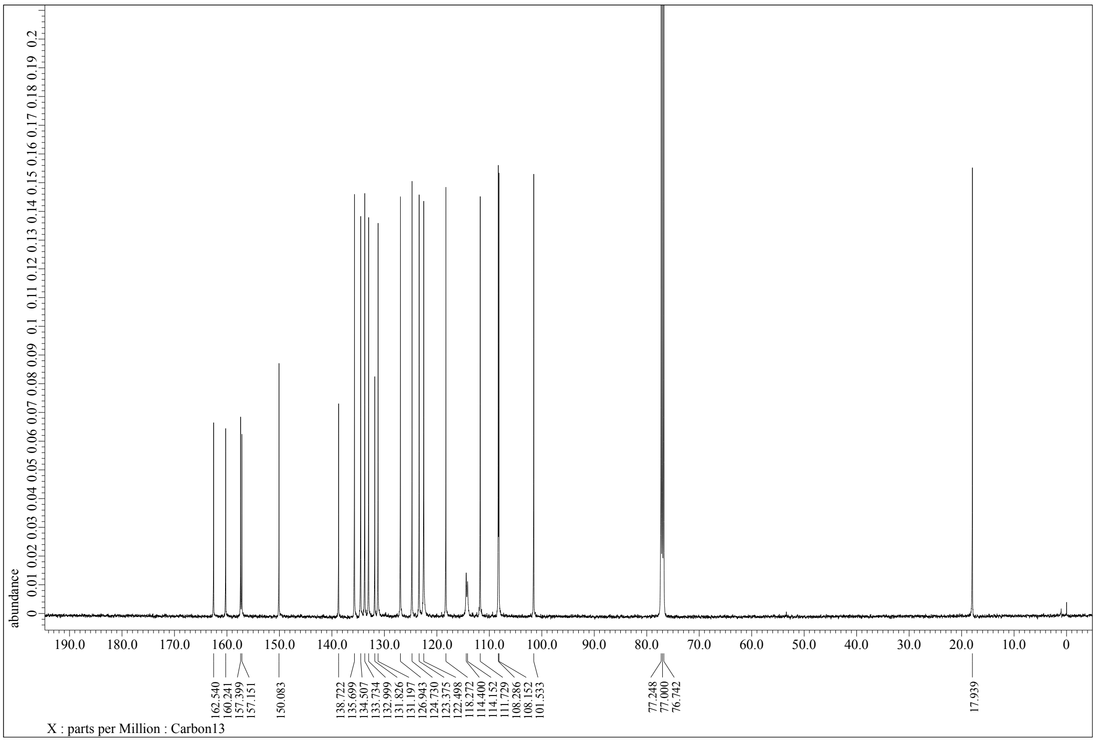

Supplementary Figure 33: **NMR data.** ^{13}C NMR spectrum of DOBNA-NH*o*Tol in $CDCl_3$ at 25 °C.

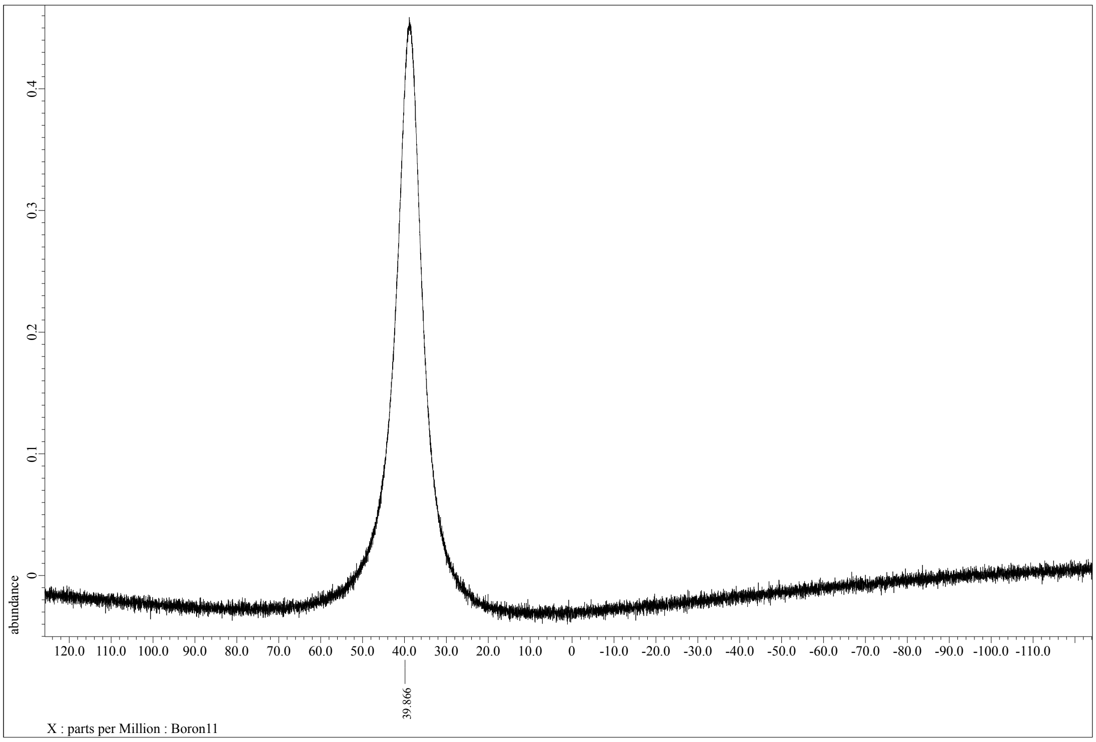

Supplementary Figure 34: **NMR data.** ^{11}B NMR spectrum of DOBNA-NH*o*Tol in $CDCl_3$ at 25 °C.

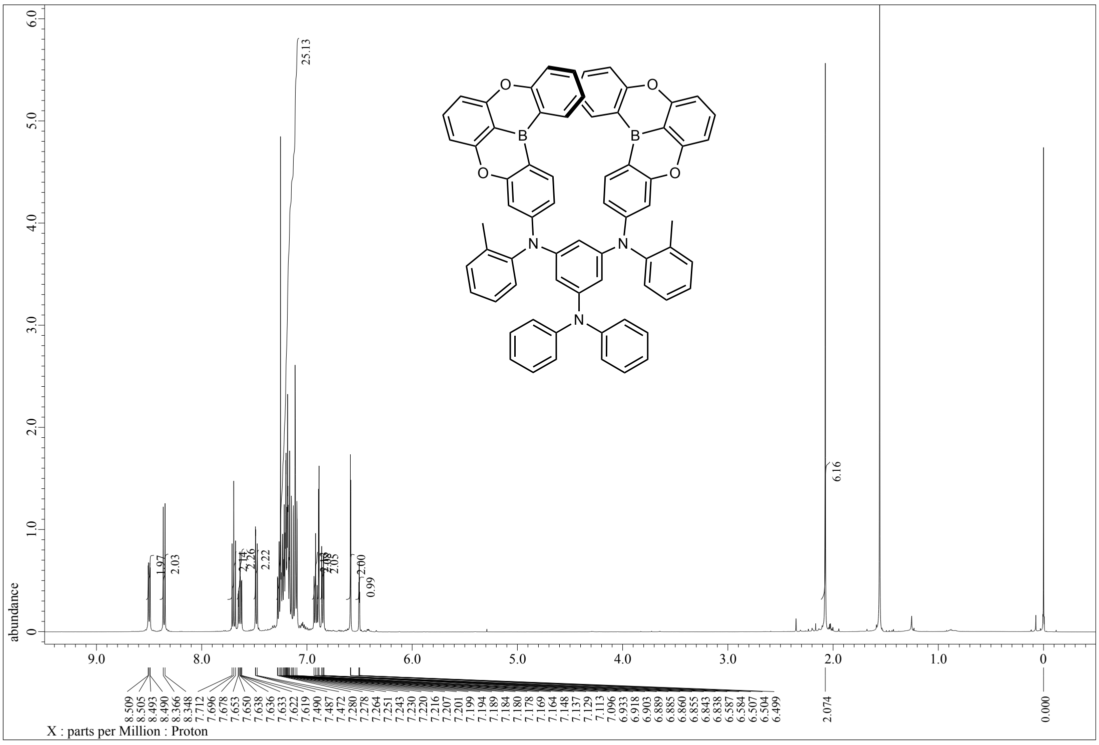

Supplementary Figure 35: **NMR data.** ^{1}H NMR spectrum of **3** in $CDCl_3$ at 25 °C.

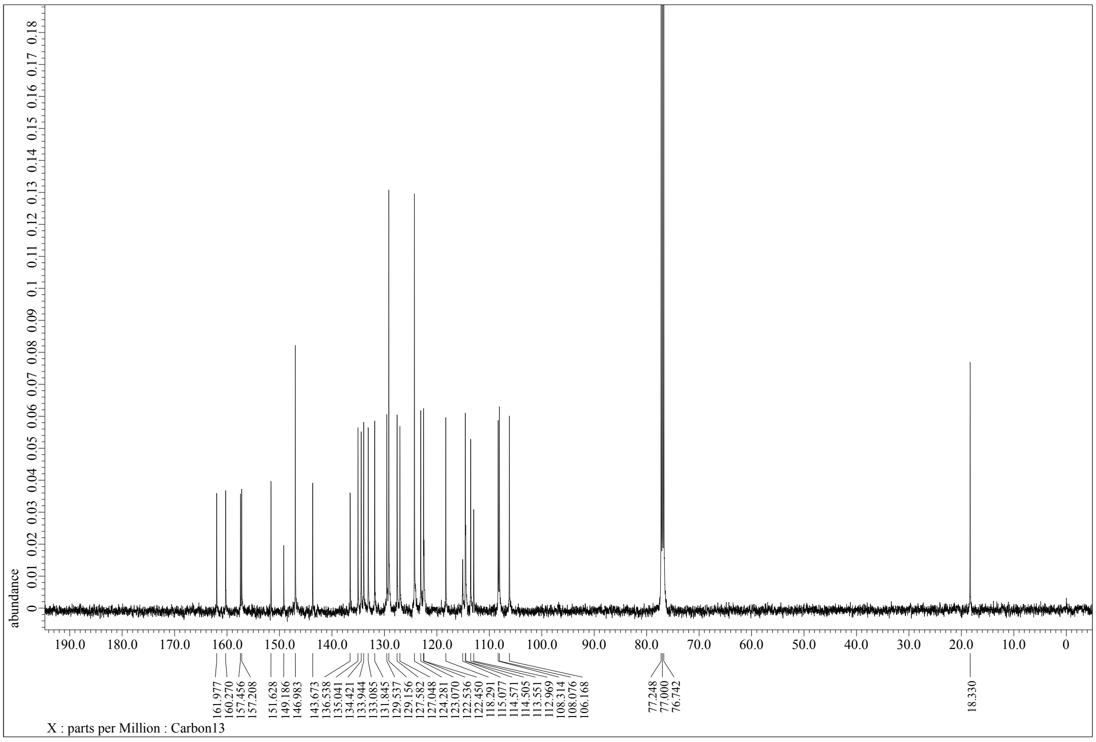

Supplementary Figure 36: **NMR data.** ^{13}C NMR spectrum of **3** in $CDCl_3$ at 25 °C.

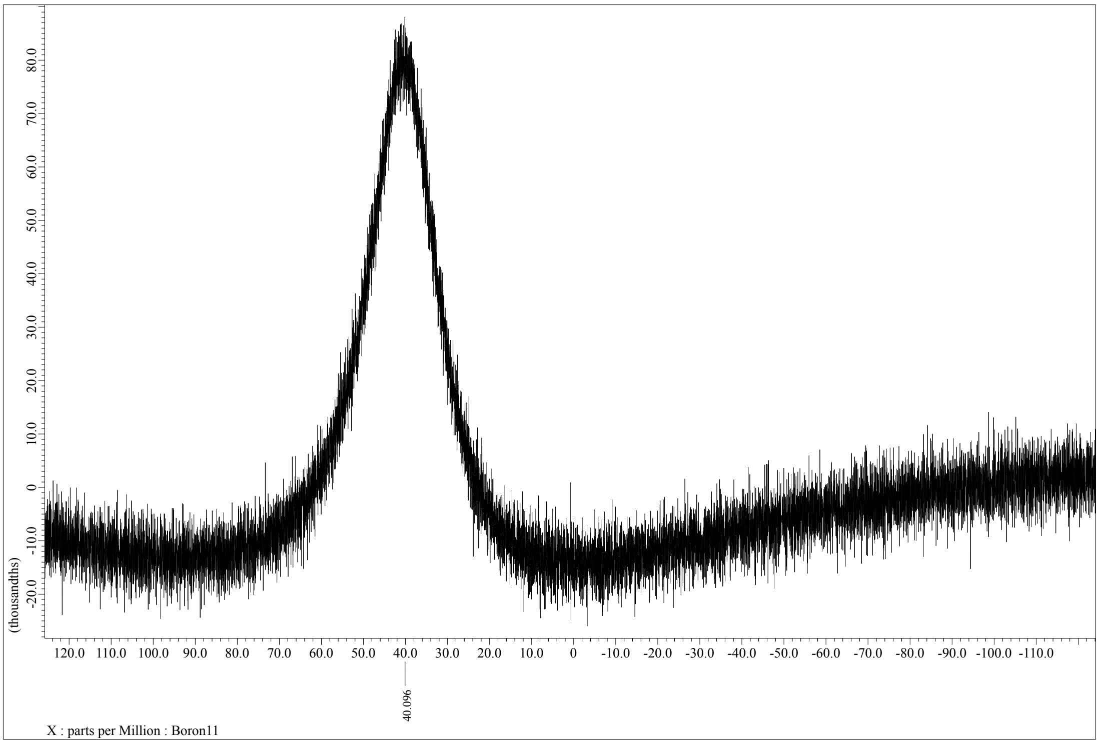

Supplementary Figure 37: **NMR data.** ^{11}B NMR spectrum of **3** in $CDCl_3$ at 25 °C.

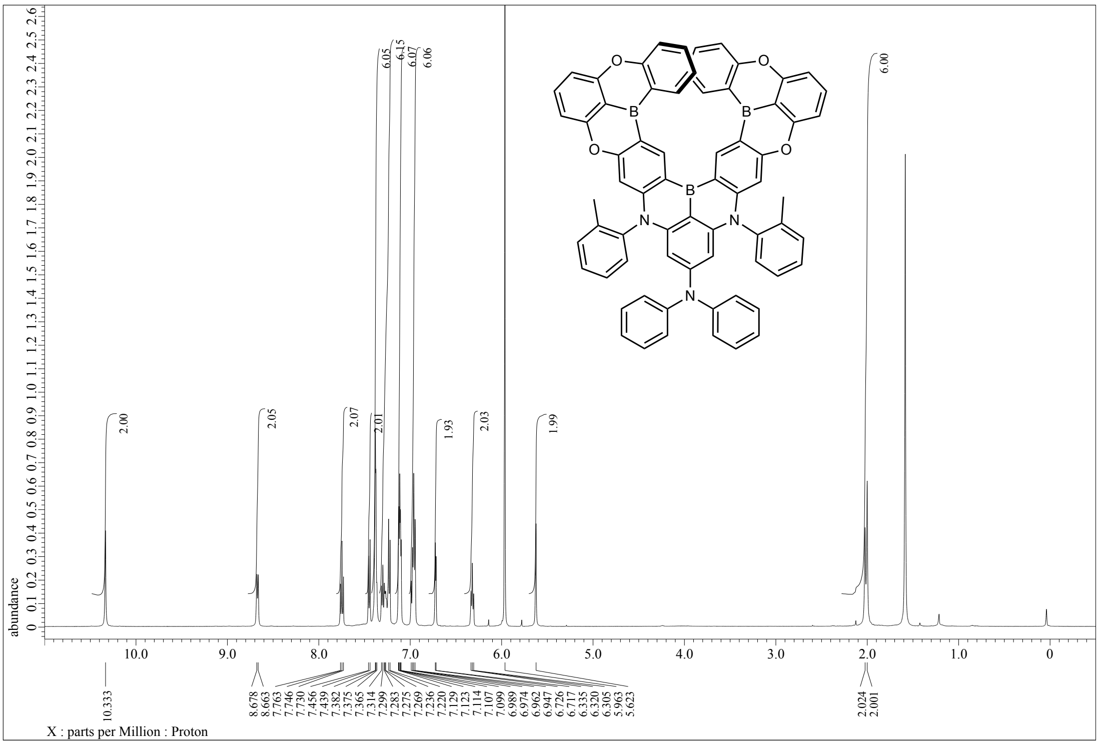

Supplementary Figure 38: **NMR data.** ^{1}H NMR spectrum of DOB2-DABNA-B-NP in tetrachloroethane–d_2 at 140 °C.

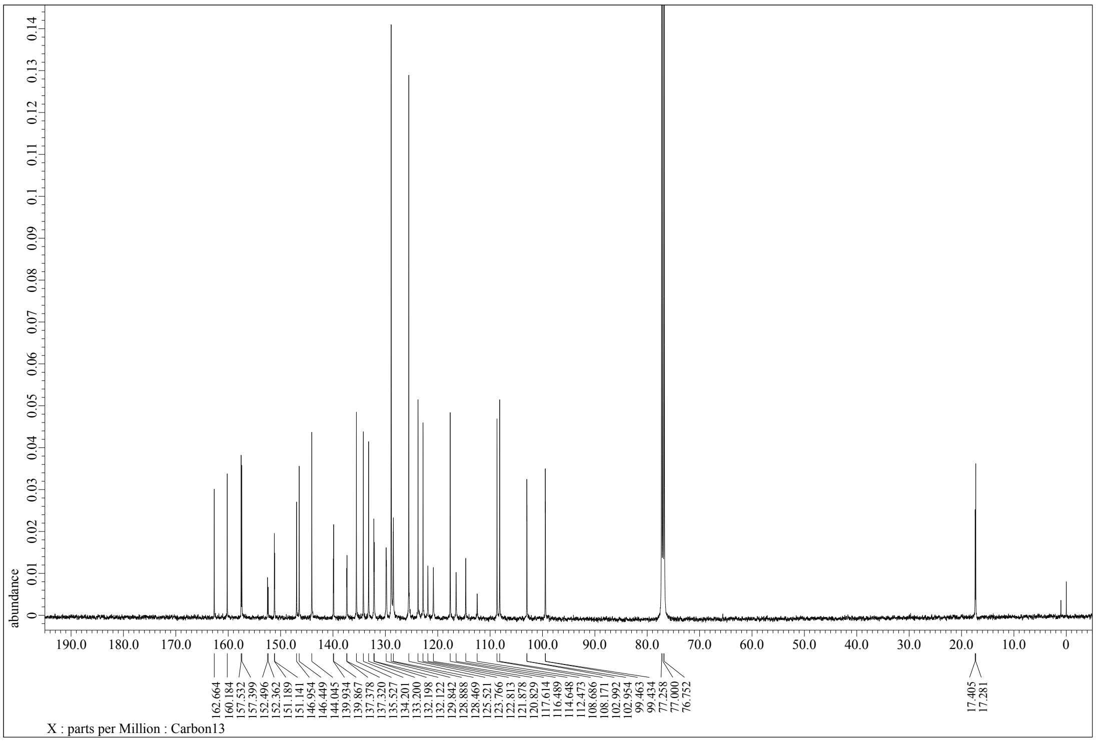

Supplementary Figure 39: **NMR data.** ^{13}C NMR spectrum of DOB2-DABNA-B-NP in $CDCl_3$ at 25 °C.

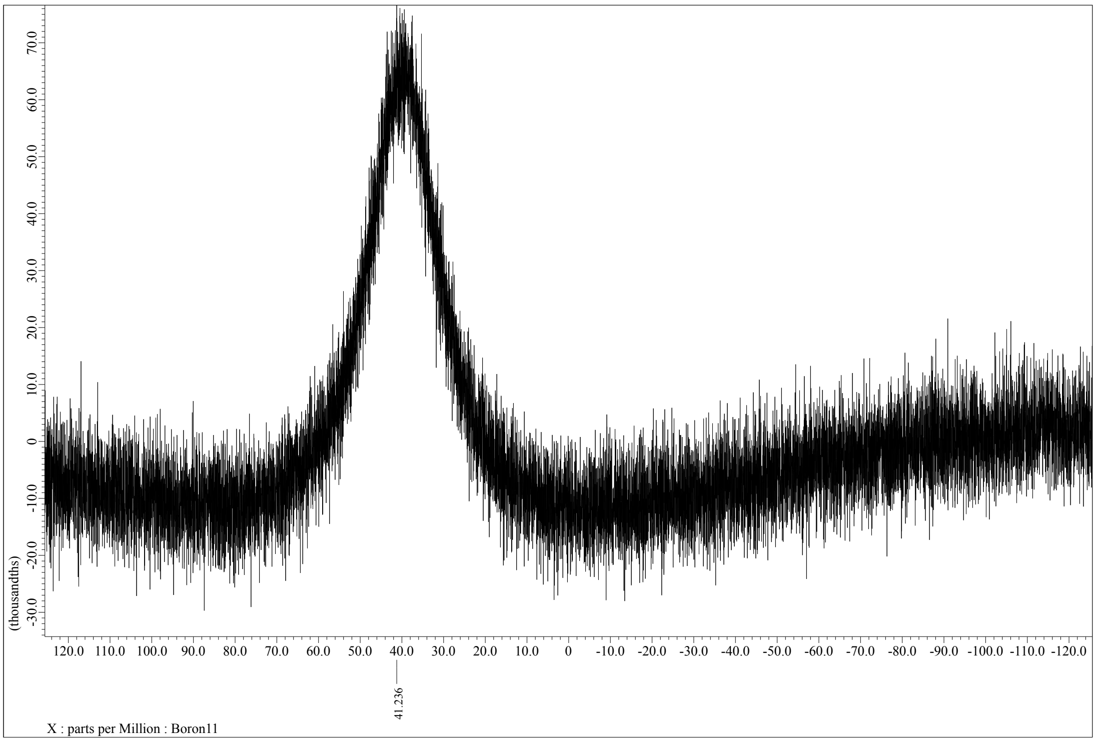

Supplementary Figure 40: **NMR data.** ^{11}B NMR spectrum of DOB2-DABNA-B-NP in $CDCl_3$ at 25 °C.

Supplementary Tables

Supplementary Table 1: Summary of TD-DFT Calculation. DOB2-DABNA-A, DOB2-DABNA-A-NP, DOB2-DABNA-B-NP, and DABNA-1 were calculated at the B3LYP/6-31G(d) Level of Theory.

compound	HOMO–1 [eV]	HOMO [eV]	LUMO [eV]	LUMO+1 [eV]	$\lambda_{(S0-S1)}$ [nm]	E_S[a] [eV]	f[b]	$\lambda_{(S0-T1)}$ [nm]	E_T[c] [eV]	ΔE_{ST}[d] [eV]
DOB2-DABNA-A	–5.19	–4.89	–1.39	–1.29	416	2.98	0.1446	448	2.77	0.21
DOB2-DABNA-A-NP	–5.02	–4.92	–1.44	–1.34	407	3.04	0.1158	452	2.74	0.30
DOB2-DABNA-B-NP	–5.05	–5.03	–1.67	–1.43	435	2.85	0.2325	478	2.60	0.25
DABNA-1	–5.59	–4.74	–1.08	–0.43	396	3.13	0.2047	470	2.64	0.49

[a]Singlet–singlet excitation energy. [b]Oscillator strength. [c]Singlet–triplet excitation energy. [d]Energy gap between S_1 and T_1 states.

compound	transition	energy	coefficient of orbital		
DOB2-DABNA-A	S_0–S_1	2.98 eV (416 nm)	HOMO → LUMO (0.68587)	HOMO → LUMO+2 (0.12970)	
	S_0–T_1	2.77 eV (448 nm)	HOMO → LUMO (0.59568)	HOMO → LUMO+2 (0.32604)	
	S_0–T_2	2.84 eV (437 nm)	HOMO–2→ LUMO+1 (0.18477)	HOMO–1 → LUMO (0.46527)	HOMO → LUMO+1 (0.39203)
			HOMO → LUMO+7 (–0.11965)		
DOB2-DABNA-A-NP	S_0–S_1	3.04 eV (407 nm)	HOMO–1 → LUMO (0.66672)	HOMO–1 → LUMO+2 (0.13672)	HOMO → LUMO+1 (0.14518)
	S_0–T_1	2.74 eV (452 nm)	HOMO–3 → LUMO (0.21808)	HOMO–2 → LUMO+1 (–0.14649)	HOMO–1 → LUMO+1 (–0.13654)
			HOMO → LUMO (0.52303)	HOMO → LUMO+2 (0.26794)	
	S_0–T_2	2.83 eV (438 nm)	HOMO–2→ LUMO (–0.18192)	HOMO–1 → LUMO (0.57843)	HOMO–1 → LUMO+2 (0.31451)
			HOMO → LUMO+1 (0.10155)		
DOB2-DABNA-B-NP	S_0–S_1	2.85 eV (435 nm)	HOMO → LUMO (0.69499)		
	S_0–T_1	2.60 eV (478 nm)	HOMO → LUMO (0.67377)	HOMO → LUMO+2 (0.13501)	
	S_0–T_2	2.71 eV (458 nm)	HOMO–4 → LUMO (–0.18276)	HOMO–3 → LUMO (–0.11034)	HOMO–1 → LUMO (0.57712)
			HOMO–1 → LUMO+2 (0.19299)	HOMO–1 → LUMO+3 (0.11525)	HOMO → LUMO+1 (–0.11415)
DABNA-1	S_0–S_1	3.13 eV (396 nm)	HOMO → LUMO (0.69744)		
	S_0–T_1	2.64 eV (470 nm)	HOMO → LUMO (0.69680)		
	S_0–T_2	3.16 eV (392 nm)	HOMO–3→ LUMO+7 (0.13191)	HOMO–2 → LUMO (0.13070)	HOMO–1 → LUMO (0.53813)
			HOMO → LUMO+5 (–0.30680)	HOMO → LUMO+7 (0.16232)	

Supplementary Table 2: Summary of Photophysical Data of DOB2-DABNA-A, DOB2-DABNA-A-NP, DOB2-DABNA-B-NP, and DABNA-1 in 1 wt%-doped PMMA film[a].

compound	λ_{ab}[b] [nm]	λ_{em}[c] [nm]	FWHM[d] [nm]	Φ[e] [%]	τ_F[f] [ns]	k_r/k_{nr}[g] [10^7 s^{-1}]	λ_{em}[h] [nm]	ΔE_{ST}[i] [meV]
DOB2-DABNA-A	434	452	27	92	6.2	8.6/0.76	452	3.6
DOB2-DABNA-A-NP	425	443	29	76	6.0	8.2/2.6	445	18
DOB2-DABNA-B-NP	453	478	28	87	3.0	10.3/1.6	479	6.5
DABNA-1	-	457	29	82	11.5	6.4/1.4	488	170

[a]Fluorescence and phosphorescence spectra of DABNA-1 was measured upon excittation at 335 nm. All other measurements were conducted upon excitation at 340 nm. [b]Maximum wavelength of absorption. [c]Maximum wavelength of fluorescence at 77 K. [d]Full width at half maximum. [e]Absolute photoluminescence quantum yield. [f]Lifetime of fluorescent (prompt) component. [g]Radiative and non-radiative rate constants. [h]Maximum wavelength of phosphorescence. [i]Energy gap between S_1 and T_1 states.

Supplementary Table 3: Summary of Transition Energies, SOC Matrix Elements, and Calculated Photophysical Data.

compound	calculation					hybrid		experiment
	SOC_{T1-S1}[a] [cm^{-1}]	E_{S1} [eV]	E_{T1} [eV]	ΔE_{S1-T1}[b] [meV]	k_{RISC}[c,d] [s^{-1}]	ΔE_{S1-T1}[e] [meV]	k_{RISC}[c,f] [s^{-1}]	k_{RISC}[e] [s^{-1}]
model-A	0.059	2.682	2.658	23	3.9×10^5	3.6[g]	8.3×10^5	1.1×10^6 [g]
						18[h]	4.7×10^5	1.2×10^6 [h]
model-B	0.035	2.529	2.449	80	1.5×10^4	6.5	2.6×10^5	3.8×10^5
DABNA-1	0.070	2.732	2.492	239	1.3×10^2	170	1.9×10^3	4.8×10^3

[a]Spin-orbit coupling between S_1 and T_1 states. [b]Energy gap between S_1 and T_1 states estimated from the calculation. [c]$k = 2\pi/\hbar g(V^2_{SOC}/\sqrt{(4\pi k_B T\lambda)})\exp(-\Delta E_{(S1-T1)}/k_B T)$. λ is assumed to be 0.10 eV. [d]Estimated by using the calculated ΔE_{S1-T1} value. [e]Spectroscopic data in PMMA films (1 wt%). [f]Estimated by using the experimental ΔE_{S1-T1} value. [g]Values of DOB2-DABNA-A. [h]Values of DOB2-DABNA-A-NP.

Supplementary Table 4: Summary of OLED Performance Employing Deep-Blue MR-TADF Emitter with CIE_y of ≤0.05.

Emitter	λ_{EL} [a] [nm]	FWHM [b] [nm]	CIE [c] (x, y)	V_{on} [d] [V]	EQE [e] [%]	CE_{max} [f] [cd A^{-1}]	PE_{max} [g] [lm W^{-1}]	LT50 [h] [h]	ref
DOB2-DABNA-A	452	24	(0.145, 0.049)	3.4	24.1/23.3/21.6/11.1	12.1	11.3	52	This work
BOBO-Z	445	18	(0.15, 0.04)	4.5	13.6/9.8/3.3/-	7.2	5.0	0.2 [i]<br>4.4 [j]	2
CzBO	448	30	(0.15, 0.05)	4.1	13.4/8.4/3.5/-	7.4	5.7	0.16	3
B-O-dpa	443	32	(0.15, 0.05)	3.8	16.3/2.2/-/-	8.3	-	(0.07) [k]	4
BisICz	437	24	(0.16, 0.04)	-	6.5/(2.0)/-/-	2.9	2.7	-	5
tBisICz	445	22	(0.16, 0.05)	-	15.1/(3.0)/-/-	8.4	8.3	-	5
tPBisICz	452	21	(0.15, 0.05)	-	23.1/(5.0)/-/-	13.5	13.3	-	5
NOBNacene	409	37	(0.173, 0.055)	4.2	8.5/-/-/-	-	-	-	6
BIC-mCz	431	38	(0.16, 0.04)	4.1	7.0/(2.0)/-/-	-	4.7	-	7
mDBIC	433	34	(0.16, 0.04)	4.1	5.7/-/-/-	-	2.0	-	7
CZCO	432	35	(0.154, 0.047)	3.4	15.6/6.0/5.9/-	8.6	7.1	-	8
1B-DTACrs	440	30	(0.154, 0.049)	10	1.31/-/-/-	-	-	-	9
2B-DTACrs	447	26	(0.150, 0.044)	4.0	14.8/10.1/-/-	-	-	-	9
*t*DIDcz	401	17	(0.164, 0.055)	4.25	2.46/-/-/-	0.39	-	-	10
gm-Icz	412	36	(0.160, 0.034)	3.75	3.04/-/-/-	0.68	-	-	10
o-Icz	433	40	(0.165, 0.046)	3.5	3.01/-/-/-	1.28	-	-	10
MesB-DIDOBNA-N	402	21	(0.170, 0.049)	4.1	16.2/3.5/-/-	-	2.7	<1.0	11

[a]Maximum wavelength of EL spectrum. [b]Full width at half maximum. [c]CIE (x, y) coordinates. [d]Turn-on voltage at the luminescence of 1 cd m^{-2}. [e]External quantum efficiency of maximum/at 100/1000/10000 cd m^{-2}. [f]Maximum current efficiency. [g]Maximum power efficiency. [h]Device half-lifetime with initial luminance of 100 cd m^{-2}. [i]In mCBP host. [j]In mCBP-CN host. [k]Initial luminescence of 10 cd m^{-2}.

Supplementary Table 5: Summary of CIE_y, EQE (@1000 cd m^{-2}), and k_{RISC} of OLEDs Employing Deep-Blue MR-TADF Emitter shown in Figures 4f and 4g.

compound	CIE_y	EQE@1000 cd m^{-2} [%]	k_{RISC} [s^{-1}]	ref
DOB2-DABNA-A	0.049	21.6	1130000	This work
BOBO-Z	0.04	3.3	70000	2
BOBS-Z	0.06	15.0	860000	2
BSBS-Z	0.08	15.9	1600000	2
CzBO	0.05	3.5	9000	3
CZCO	0.047	5.9	-	8
2B-DTACrs	0.044	-	130000	9
CzBNO	0.08	5.0	34700	12
BN1	0.06	3.1	13000	13
BN3	0.07	6.6	255000	13
t-DAB-DPA	0.08	9.2	39700	14
4F-ν-DABNA	0.08	-	228000	15
4F-m-ν-DABNA	0.06	-	210000	15
TPD4PA	0.06	17.8	251000	16
tBu-tPD4PA	0.07	20.5	244000	16
tDPAC-BN	0.094	5.4	11600	17
PAB	0.076	-	69500	18
2tPAB	s0.076	2.1	63400	18
3tPAB	0.076	2.1	57400	18
BSS-Cz	0.07	-	150000	19
BFCz-DABNA	0.09	-	27800	20

Supplementary Table 6: **Ionization Potential (I_p), Optical Band Gap (E_g), and Electron Affinity (E_a) of DOB2-DABNA-A and DOB2-DABNA-B-NP.**

compound	HOMO [eV]	I_p [eV]	E_g [eV]	E_a [eV]
DABNA-1[a]	–4.74	5.58	2.67	2.91
DOB2-DABNA-A	–4.89	5.73[b]	2.74[c]	2.99[d]
DOB2-DABNA-B-NP	–5.03	5.87[b]	2.62[c]	3.25[d]

[a]ref[1]. [b]Estimated from I_p of DABNA-1 and HOMO energy levels. [c]Estimated from the onset wavelength of the UV-vis absorption spectrum in 1 wt% PMMA film. [d]Calculated from I_p and E_g.

Supplementary Methods

Synthesis of *N*–phenyl–5,9–dioxa–13b–boranaphtho[3,2,1–*de*]anthracen–7–amine (DOBNA-NHPh)

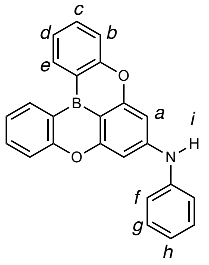

SPhos (90.5 mg, 0.22 mmol), tris(dibenzylideneacetone)dipalladium(0) (92.0 mg, 0.10 mmol), 7-chloro-5,9-dioxa-13b-boranaphtho[3,2,1-*de*]anthracene (1.52 g, 5.0 mmol), potassium *tert*-butoxide (1.14 g, 10 mmol), and aniline (0.55 mL, 6.0 mmol) were dissolved in toluene (40 mL) under a nitrogen atmosphere. After stirring at 80 °C for 40 h, the reaction mixture was filtered with a pad of silica gel (eluent: dichloromethane). After the filtrate was condensed *in vacuo* to dryness, the crude product was washed with methanol to obtain the title compound as a colorless solid (1.01 g, 61% yield, 98% pure on ^{1}H NMR analysis). IR (neat): cm^{-1} 3370, 3076, 3036 (Ar–H), 2359, 2336, 1628, 1591, 1580, 1516, 1495, 1489, 1474, 1456, 1443, 1404, 1344, 1314, 1294, 1279, 1244, 1215, 1209, 1157, 1142, 1065, 816, 754, 727, 692, 656; mp: 218.5–220.0 ºC; ^{1}H NMR (500 MHz, $CDCl_3$) δ 6.20 (brs, 1H, *i*), 6.78 (s, 2H, *a*), 7.14 (t, *J* = 7.5 Hz, 1H, *h*), 7.30 (dd, *J* = 7.5 Hz, 1.0 Hz, 2H, *f*), 7.34 (t, *J* = 7.5 Hz, 2H, *d*), 7.40 (t, *J* = 7.5 Hz, 2H, *g*), 7.45 (d, *J* = 7.5 Hz, 2H, *b*), 7.64 (t, *J* = 7.5 Hz, 2H, *c*), 8.62 (dd, *J* = 7.5 Hz, 2.0 Hz, 2H, *e*); ^{13}C NMR (126 MHz, $CDCl_3$) δ 95.4 (2C), 118.2 (2C), 121.5 (2C), 122.6 (2C), 123.7 (1C), 129.6 (2C), 132.8 (2C), 134.4 (2C), 140.9 (1C), 150.9 (1C), 159.2 (2C), 160.7 (2C). The NMR signal of the carbon α to the boron was not observed.; ^{11}B NMR (160 MHz, $CDCl_3$) δ 38.6. HRMS (MALDI) *m/z* $[M]^+$ calcd for $C_{24}H_{16}BNO_2$ 361.1274, observed 361.1290.

Synthesis of N^1,N^3-di(5,9-dioxa-13b-boranaphtho[3,2,1-*de*]anthracen-7-yl)-N^1,N^3,N^5,N^5-tetra phenylbenzene-1,3,5-triamine (1)

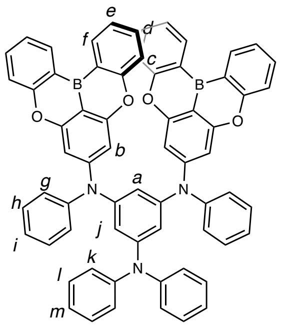

3,5-Dichloro-*N*,*N*-diphenylaniline (0.126 g, 0.40 mmol) was added to a mixture of DOBNA-NHPh (0.303 g, 0.84 mmol), tris(dibenzylideneacetone)palladium(0) (15.1 mg, 0.016 mmol), tri-*tert*-

butylphosphonium tetrafluoroborate (18.1 mg, 0.064 mmol) and potassium *tert*-butoxide (0.134 g, 1.2 mmol) in toluene (6.0 mL) under a nitrogen atmosphere. After stirring at 120 ºC for 24 h, the reaction mixture was condensed *in vacuo* to dryness and then washed with water by using a sonicator. The crude product was washed with hexane to obtain the title compound (0.282 g, 73% yield, 97% pure on ^{1}H NMR analysis) as a white solid. IR (neat): cm^{-1} 3074, 3034, (Ar–H), 2363, 1942, 1626, 1570, 1439, 1294, 1221, 1063, 752, 694; mp: >300 ºC, ^{1}H NMR (500 MHz, tetrachloroethane–d_2) δ 6.65 (s, 1H, *a*), 6.72 (d, *J* = 2.0 Hz, 2H, *j*), 6.79 (s, 4H, *b*), 6.88 (t, *J* = 7.5 Hz, 2H, *m*), 7.12–7.14 (m, 6H, *i*,*k*), 7.18 (t, *J* = 7.5 Hz, 4H, *l*), 7.26 (d, *J* = 7.0 Hz, 4H, *g*), 7.33–7.37 (m, 8H, *e*,*h*), 7.46 (d, *J* = 8.0 Hz, 4H, *c*), 7.65 (td, *J* = 8.0 Hz, 1.0 Hz, 4H, *d*), 8.62 (dd, *J* = 8.0 Hz, 1.0 Hz, 4H, *f*); ^{13}C NMR peaks were barely detected because of the low solubility of the title compound. ^{11}B NMR (160 MHz, tetrachloroethane–d_2) δ 38.0. HRMS (MALDI) *m/z* [M]$^{+}$ calcd for $C_{66}H_{43}B_2N_3O_4$ 963.3440, observed 963.3436.

Synthesis of *N*,*N*,11,15-tetraphenyl-11,15-dihydro-9,17,26,27-tetraoxa-11,15-diaza-4b,21b,26c-triboranaphtho[3,2,1-*hi*]naphtho[1',2',3':11,12]tetraceno[3,2,1-*de*]pentacene-13-amine (DOB2-DABNA-A-NP)

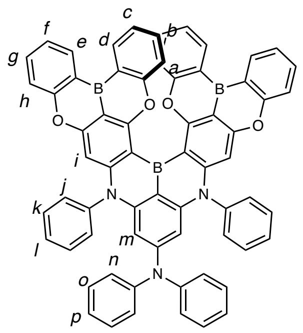

Compound **1** (0.097 g, 0.10 mmol), boron triiodide (0.315 g, 0.80 mmol) and 2,6-di-*tert*-butylpyridine (0.130 mL, 0.60 mmol) were dissolved in toluene (2.5 mL) at 0 °C under a nitrogen atmosphere. After stirring at rt for 19 h, reaction mixture was diluted by dichloromethane and quenched by phosphate buffer solution at 0 °C. The aqueous layer was separated and extracted with dichloromethane. The combined organic layer was washed by saturated $Na_2S_2O_3$ aqueous solution and removed *in vacuo*. The resulting crude product was dissolved in toluene (2.0 mL) and acetic acid (0.115 mL, 2.01 mmol) at room temperature. After stirring at 100 °C for 1 h, saturated sodium carbonate was added to the reaction mixture, and then the aqueous layer was extracted with dichloromethane. The yield of the title compound in the crude product was determined to be 42% yield by ^{1}H NMR analysis using dibromomethane as an internal standard. The crude product was purified by silica gel column chromatography (eluent: hexane/dichloromethane = 92/8 ~ 34/66 (gradient)). to obtain 52.1 mg semi-purified product. After that, 6.5 mg from the 52.1 mg was separated and purified by HPLC to obtain the title compound (3.4 mg, 98.5% pure on HPLC analysis) as a yellow solid. Therefore, the total

isolate yield is estimated to be 28%. IR (neat): cm^{-1} 3517, 2916 (Ar–H), 2359, 2330, 1950, 1800, 1732, 1568, 1489, 1456, 1298, 756, 733, 696, 623; mp: >300 ºC, ^{1}H NMR (500 MHz, $CDCl_3$) δ 5.84 (s, 2H, *m*), 6.38 (dd, *J* = 7.5 Hz, 1.0 Hz, 2H, *a*), 6.55 (s, 2H, *i*), 6.91–6.99 (m, 10H, *b,c,n,p*), 7.12 (t, *J* = 7.5 Hz, 4H, *o*), 7.35 (td, *J* = 7.5 Hz, 1.0 Hz, 2H, *f*), 7.39 (d, *J* = 7.5 Hz, 4H, *j*), 7.40–7.48 (m, 4H, *h,l*), 7.57 (t, *J* = 7.5 Hz, 4H, *k*), 7.65 (td, *J* = 7.5 Hz, 1.0 Hz, 4H, *g*), 8.43 (dd, *J* = 7.5 Hz, 1.0 Hz, 2H, *d*), 8.64 (dd, *J* = 7.5 Hz, 1.0 Hz, 2H, *e*); ^{13}C NMR (126 MHz, $CDCl_3$) δ 96.0 (2C), 100.6 (2C), 108.6 (1C), 108.9 (1C), 113.5 (1C), 117.9 (2C), 118.1 (2C), 122.0 (2C), 122.5 (1C), 122.6 (2C), 123.0 (1C), 123.6 (2C), 125.5 (8C), 128.5 (2C), 128.9 (8C), 130.2 (2C), 130.9 (2C), 132.3 (2C), 132.9 (2C), 133.2 (2C), 134.6 (2C), 141.8 (2C), 146.6 (2C), 146.7 (2C), 150.8 (1C), 153.3 (2C), 159.3 (2C), 160.5 (2C+2C), 163.4 (2C); ^{11}B NMR (160 MHz, $CDCl_3$) δ 39.3; HRMS (MALDI) *m/z* $[M]^+$ calcd for $C_{66}H_{40}B_3N_3O_4$ 971.3321, observed 971.3301.

Synthesis of *N*-(3,5-dimethylphenyl)-5,9-dioxa-13b-boranaphtho[3,2,1-*de*]anthracen-7-amine (DOBNA-NHxyl)

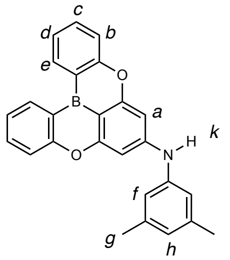

3,5-Dimethylaniline (1.50 mL, 12 mmol) was added to a mixture of 7-chloro-5,9-dioxa-13b-boranaphtho[3,2,1-*de*]anthracene (3.05 g, 10 mmol), tris(dibenzylideneacetone)dipalladium(0) (0.183 g, 0.20 mmol), SPhos (0.186 g, 0.44 mmol), and potassium *tert*-butoxide (4.28 g, 20 mmol) in toluene (100 mL) under a nitrogen atmosphere. After stirring at 70 ºC for 44 h, the reaction mixture was filtered with a pad of silica gel (eluent: dichloromethane). After the filtrate was condensed *in vacuo*, the crude product was purified by silica gel column chromatography (eluent: hexane/dichloromethane = 5/1, 3/1) to obtain the title compound (3.40 g, 87% yield, 97% pure on ^{1}H NMR analysis) as a white solid. IR (neat): cm^{-1} 3354 (N–H), 3075, 2918 (Ar–H), 2361, 1587, 1441, 1339, 1219, 1056, 824, 694; mp: 183.0–184.0°C, ^{1}H NMR (500 MHz, $CDCl_3$) δ 2.35 (s, 6H, *g*), 6.11 (brs, 1H, *k*), 6.75 (s, 2H, *a*), 6.78 (s, 1H, *h*), 6.92 (s 2H, *f*), 7.34 (t, *J* = 7.5 Hz, 2H, *d*), 7.45 (t, *J* = 7.5 Hz, 2H, *b*), 7.64 (t, *J* = 7.5 Hz, 2H, *c*), 8.62 (d, *J* = 7.5 Hz, 2H, *e*); ^{13}C NMR (126 MHz, $CDCl_3$) δ 21.4 (2C), 95.1 (2C), 108.8 (2C), 118.0 (2C), 119.0 (2C), 122.5 (2C), 125.4 (1C), 132.7 (2C), 134.2 (2C), 139.2 (2C), 140.4 (1C), 150.7 (1C), 158.8 (2C), 160.4 (2C). The NMR signal of the carbon α to the boron was not observed.; ^{11}B NMR (160 MHz, $CDCl_3$) δ 38.7. HRMS (MALDI) *m/z* $[M]^+$ calcd for $C_{25}H_{18}BNO_2$ 375.1431, observed 375.1418.

Synthesis of *N*^{1},*N*^{3}-di(5,9-dioxa-13b-boranaphtho[3,2,1-*de*]anthracen-7-yl)-5-(*tert*-butyl)-*N*^{1},*N*^{3}-

bis(3,5-dimethylphenyl)benzene-1,3-diamine (2)

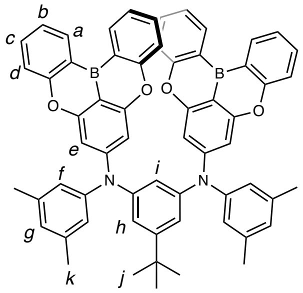

1,3-Dibromo-5-*tert*-butylbenzne (0.359 g, 1.2 mmol), DOBNA-NHxyl (0.961 g, 2.5 mmol), tris(dibenzylideneacetone)dipalladium(0) (54.1 mg, 0.059 mmol), tri-t*ert*-butylphosphonium tetrafluoroborate (54.9 mg, 0.19 mmol) and potassium *tert*-butoxide (0.414 g, 3.7 mmol) in toluene (12 mL) under a nitrogen atmosphere. After stirring at 80 ºC for 14 h, the reaction mixture was allowed to cool to room temperature. After the reaction mixture was filtered with a pad of silica gel (eluent: dichloromethane), the solvent was removed *in vacuo*. The crude product was purified by silica gel column chromatography (eluent: hexane/dichloromethane = 94/6 ~ 50/50 (gradient)) and washed with acetonitrile to obtain the title compound (0.576 g, 63% yield, 99% pure on ^{1}H NMR analysis) as a pale yellow solid. IR(neat): cm^{-1} 3072, 3035, 2954, 2917, 2864, 2707, 2375, 2292, 2057, 2000, 1940, 1626, 1572, 1543, 1490, 1473, 1439, 1412, 1319, 1290, 1253, 1218, 1184, 1129, 1098, 1064, 1039, 945, 925, 851, 828, 749, 723, 697; mp: 212.5-241.3 °C; ^{1}H NMR (495 MHz, $CDCl_3$) δ 1.25 (s, 9H, *j*), 2.28 (s, 12H, *k*), 6.75 (s, 4H, *e*), 6.79 (s, 2H, *g*), 6.89 (s, 4H, *f*), 6.92 (t, *J* = 2.0 Hz, 1H, *i*), 7.08 (d, *J* = 1.7 Hz, 2H, *h*), 7.33 (ddd, *J* = 0.85, 7.2, 7.5 Hz, 4H, *b*), 7.40 (dd, *J* = 0.57, 7.9 Hz, 4H, *d*), 7.61(ddd, *J* = 1.4, 7.7, 7.7 Hz, 4H, *c*), 8.61 (dd, *J* = 1.4, 7.5 Hz, 4H, *a*); ^{13}C NMR (125 MHz, $CDCl_3$) δ 21.4 (4C), 31.3 (3C), 35.1 (1C), 100.2 (4C), 109.6 (2C), 118.2 (4C), 119.4 (4C), 119.8(2C), 121.2 (1C), 122.6 (4C), 124.4 (4C), 127.0 (2C), 133.0 (4C), 134.4 (4C), 139.3 (4C), 146.3 (2C), 147.3 (2C), 154.0 (1C), 154.5 (2C), 158.5 (4C), 160.7 (4C); ^{11}B NMR (159 MHz, $CDCl_3$) δ 37.7; HRMS (MALDI) *m/z* [M]$^{+}$ calcd. for $C_{62}H_{50}B_2N_2O_4$ 908.3957; observed 908.3959.

Synthesis of 13-(*tert*-butyl)-11,15-bis(3,5-dimethylphenyl)-11,15-dihydro-9,17,26,27-tetraoxa-11,15-diaza-4b,21b,26c-triboranaphtho[3,2,1-*hi*]naphtho[1',2',3':11,12]tetraceno[3,2,1-*de*] pentacene (DOB2-DABNA-A)

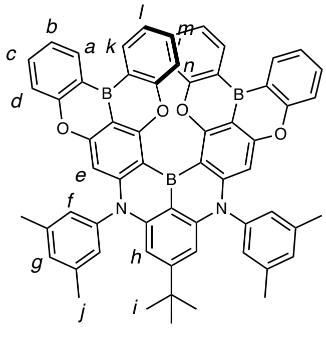

Compound **2** (0.277 g, 0.30 mmol), boron triiodide (0.941 g, 2.4 mmol) and 2,6-di-*tert*-butylpyridine (0.400 mL, 1.8 mmol) were dissolved in toluene (7.5 mL) at 0 °C under a nitrogen atmosphere. After stirring at rt for 19 h, reaction mixture was diluted by dichloromethane and quenched by phosphate buffer solution at 0 °C. The aqueous layer was separated and extracted with dichloromethane. The combined organic layer was washed by saturated $Na_2S_2O_3$ aqueous solution and removed *in vacuo*. The yield of the title compound in the crude product was determined to be 40% yield by ^{1}H NMR analysis using tetrachloroethane as an internal standard. The resulting crude product was purified by silica gel column chromatography (eluent: hexane/dichloromethane = 92/8 ~ 34/66 (gradient)) to obtain the title compound (0.066 g, 24% yield, >99.5% pure on HPLC analysis) as a yellow solid. IR(neat): cm^{-1} 2961, 2873, 2194, 2182, 1621, 1597, 1569, 1457, 1411, 1354, 1337, 1298, 1251, 1219, 1201, 1158, 1063, 841, 758, 733, 707, 689, 663; mp: 311.3–332.6 °C; ^{1}H NMR (495 MHz, $CDCl_3$) δ 1.08 (s, 9H, *i*), 2.51 (s, 12H, *j*), 6.39 (d, *J* = 8.2 Hz, 2H, *n*), 6.42 (s, 2H, *h*), 6.67 (s, 2H, *e*), 6.93 (t, *J* = 7.1 Hz, 2H, *m*), 6.97 (t, *J* = 7.4 Hz, 2H, *l*), 7.17 (br, 4H, *f*), 7.31 (s, 2H, *g*), 7.36 (t, *J* = 7.1 Hz, 2H, *b*), 7.49 (d, *J* = 8.2 Hz, 2H, *d*), 7.66 (t, *J* = 7.1 Hz, 2H, *c*), 8.43 (d, *J* = 7.1 Hz, 2H, *k*), 8.65 (d, *J* = 7.1 Hz, 2H, *a*); ^{13}C NMR (125 MHz, $CDCl_3$) δ 21.6 (4C), 31.2 (3C), 35.6 (1C), 96.1 (2C), 104.9 (2C), 108.8 (2C), 108.9 (2C), 116.3 (1C), 118.0 (2C), 118.3 (2C), 122.1 (2C), 122.7 (2C), 123.1 (2C), 127.4 (4C), 128.3 (4C), 130.5 (2C), 132.3 (2C), 133.0 (2C), 133.3 (2C), 134.8 (2C), 140.3 (2C), 141.5 (2C), 141.9 (2C), 145.6 (2C), 153.4 (2C), 154.8 (1C), 159.4 (2C), 160.6 (2C), 163.7 (2C); ^{11}B NMR (159 MHz, $CDCl_3$) δ 36.7. HRMS (MALDI) *m/z* $[M]^+$ calcd. for $C_{62}H_{47}B_3N_2O_4$ 916.3815; observed 916.3820.

Synthesis of *N*-(*o*-tolyl)-5,9-dioxa-13b-boranaphtho[3,2,1-*de*]anthracen-3-amine (DOBNA-NH*o*Tol)

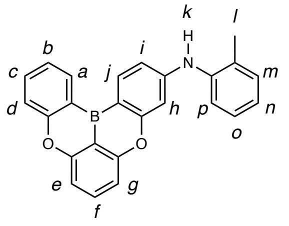

o-Toluidine (0.643 mL, 6.0 mmol) was added to a mixture of 3-chloro-5,9-dioxa-13b-boranaphtho[3,2,1-*de*]anthracene (1.54 g, 5.0 mmol), tris(dibenzylideneacetone)dipalladium(0) (91.7 mg, 0.10 mmol), 2-dicyclohexylphosphino-2’,6’-dimethoxybiphenyl (SPhos, 89.8 mg, 0.22 mmol), and potassium *tert*-butoxide (1.12 g, 10 mmol) in toluene (25 mL) under a nitrogen atmosphere. After stirring at 70 ºC for 20 h, the reaction mixture was filtered with a pad of silica gel (eluent: toluene). After the filtrate was condensed *in vacuo*, the crude product was purified by silica gel column chromatography (eluent: hexane/dichloromethane = 10/1, 5/1) to obtain the title compound (1.69 g, 90% yield, 97% pure on ^{1}H NMR analysis) as a white solid. IR (neat): cm^{-1} 3412, (N–H), 3069, 3036

(Ar–H), 2311, 1611, 1589, 1579, 1456, 1427, 1344, 1290, 1254, 1227, 1043, 785, 748; mp: 137.0–137.5 °C, ^{1}H NMR (500 MHz, $CDCl_3$) δ 2.32 (s, 3H, *l*), 5.81 (brs, 1H, *k*), 6.84 (d, *J* = 2.5 Hz, 1H, *h*), 6.86 (dd, *J* = 8.0 Hz, 2.5 Hz, 1H, *i*), 7.12 (d, *J* = 8.0 Hz, 1H, *g*), 7.13 (t, *J* = 7.5 Hz, 1H, *n*), 7.16 (d, *J* = 8.0 Hz, 1H, *e*), 7.26 (t, *J* = 7.5 Hz, 1H, *o*), 7.30 (d, *J* = 7.5 Hz, 1H, *m*), 7.34 (td, *J* = 8.0 Hz, 1.5 Hz, 1H, *b*), 7.41 (d, *J* = 7.5 Hz, 1H, *p*), 7.50 (dd, *J* = 8.0 Hz, 1.5 Hz, 1H, *d*), 7.64–7.71 (m, 2H, *c*,*f*), 8.50 (d, *J* = 8.0 Hz, 1H, *j*), 8.60 (dd, *J* = 8.0 Hz, 1.5 Hz, 1H, *a*); ^{13}C NMR (126 MHz, $CDCl_3$) δ 17.9 (1C), 101.5 (1C), 108.2 (1C), 108.3 (1C), 111.7 (1C), 114.2–114.4 (3C), 118.3 (1C), 122.5 (1C), 123.4 (1C), 124.7 (1C), 126.9 (1C), 131.2 (1C), 131.9 (1C), 133.0 (1C), 133.7 (1C), 134.5 (1C), 135.7 (1C), 138.7 (1C), 150.1 (1C), 157.2 (1C), 157.4 (1C), 160.2 (1C), 162.5 (1C); ^{11}B NMR (160 MHz, $CDCl_3$) δ 39.9. HRMS (MALDI) *m/z* $[M]^+$ calcd for $C_{26}H_{20}BNO_2$ 389.1587, observed 389.1583.

Synthesis of *N*^{1},*N*^{3}-di(5,9-dioxa-13b-boranaphtho[3,2,1-*de*]anthracen-11-yl)-*N*^{5},*N*^{5}-diphenyl-*N*^{1},*N*^{3}-di-*o*-tolylbenzene-1,3,5-triamine (3)

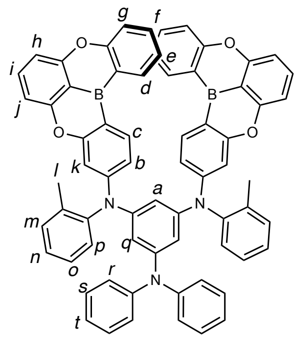

3,5-Dibromo-*N*,*N*-diphenylaniline (0.405 g, 1.0 mmol) was added to a mixture of DOBNA-NH*o*Tol (0.789 g, 2.1 mmol), tris(dibenzylideneacetone)dipalladium(0) (46.0 mg, 0.050 mmol), tri-*tert*-butylphosphonium tetrafluoroborate (43.6 mg, 0.15 mmol) and potassium *tert*-butoxide (0.336 g, 3.0 mmol) in toluene (10 mL) under a nitrogen atmosphere. After stirring at 80 ºC for 38 h, the reaction mixture was condensed *in vacuo* to dryness and then washed with water by using a sonicator. The crude product was washed with hexane to obtain the title compound (0.686 g, 69% yield, 97% pure on ^{1}H NMR analysis) as a yellow solid. IR (neat): cm^{-1} 3064, 2922 (Ar–H), 2364, 2345, 1607, 1573, 1456, 1292, 1097, 1040, 789, 750, 694; mp: >300 ºC, ^{1}H NMR (500 MHz, $CDCl_3$) δ 2.07 (s, 6H, *l*), 6.50 (t, *J* = 1.5 Hz, 1H, *a*), 6.59 (d, *J* = 1.5 Hz, 2H, *q*), 6.85 (dd, *J* = 8.5 Hz, 2.5 Hz, 2H, *b*), 6.89 (d, *J* = 2.5 Hz, 2H, *k*), 6.92 (t, *J* = 7.5 Hz, 2H, *t*), 7.01–7.23 (m, 22H, *e*,*h*,*j*,*m*,*n*,*o*,*p*,*r*,*s*), 7.48 (d, *J* = 8.0 Hz, 2H, *g*), 7.64 (td, *J* = 1.5 Hz, 8.0 Hz, 2H, *f*), 7.70 (d, *J* = 8.0 Hz, 2H, *i*), 8.36 (d, *J* = 8.5 Hz, 2H, *c*), 8.50 (dd, *J* = 8.0 Hz, 1.5 Hz, 2H, *d*); ^{13}C NMR (126 MHz, $CDCl_3$) δ 18.3 (2C), 106.2 (2C), 108.1 (2C), 108.3 (2C), 113.0 (1C), 113.6 (2C), 114.5 (2C), 114.6 (2C), 115.1 (2C), 118.3 (2C), 122.5 (2C+2C), 123.1 (2C), 124.3 (4C), 127.0 (2C), 127.6 (2C), 129.2 (4C), 129.5 (2C), 131.8 (2C), 133.1 (2C), 133.9 (2C), 134.4 (2C), 135.0 (2C), 136.5 (2C), 143.7 (2C), 147.0 (2C+2C), 149.2 (1C), 151.6 (2C), 157.2 (2C), 157.5 (2C), 160.3 (2C), 162.0 (2C); ^{11}B NMR (160 MHz, $CDCl_3$) δ 40.1. HRMS

(MALDI) *m/z* [M]$^+$ calcd for $C_{68}H_{47}B_2N_3O_4$ 991.3753, observed 991.3778.

Synthesis of *N,N*-diphenyl-11,15-di-*o*-tolyl-11,15-dihydro-5,9,17,21-tetraoxa-11,15-diaza-25b,26b,27b-triboranaphtho[3,2,1-*de*]naphtho[3',2',1':10,11]tetraceno[1,2,3-*jk*]pentacen-13-amine (DOB2-DABNA-B-NP)

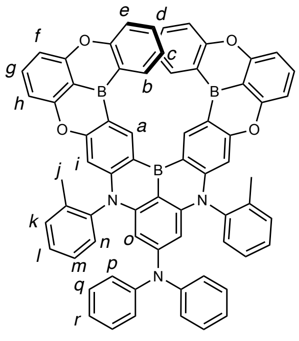

Boron tribromide (0.456 mL, 4.8 mmol) was added to a solution of **3** (0.298 g, 0.30 mmol) in chlorobenzene (6.0 mL) under a nitrogen atmosphere. After stirring at 150 ºC for 20 h, the reaction mixture was allowed to cool to room temperature and hydrogen bromide was removed *in vacuo*. After phosphorus buffer solution (pH = 7, 10 mL) was added to the reaction mixture at 0 ºC, the aqueous layer was separated and extracted with dichloromethane (100 mL, three times). After the combined organic layer was condensed *in vacuo*, the crude product was dissolved in toluene (6.0 mL) and acetic acid (0.343 mL, 6.00 mmol) at room temperature. After stirring at 100 °C for 1 h, saturated sodium carbonate was added to the reaction mixture, and then the aqueous layer was extracted with dichloromethane. The yield of the title compound in the crude product was determined to be 41% yield by ^{1}H NMR analysis using tetrachloroethane as an internal standard. The crude product was purified by GPC (eluent: 1,2-dichloroethane) and washed with heptane and acetonitrile to obtain the title compound (37.6 mg, 12% yield, 97% pure on HPLC analysis) as a yellow solid. IR (neat): cm^{-1} 3063, 2951 (Ar–H), 1580, 1429, 1294, 1246, 787, 733, 696; mp: >300 ºC, ^{1}H NMR (500 MHz, tetrachloroethane–d_2) δ 2.01–2.02 (m, 6H, *j*), 5.62 (s, 2H, *o*), 6.32 (t, *J* = 7.5 Hz, 2H, *c*), 6.72 (m, 1H, *i*), 6.95–6.99 (m, 6H, *p*,*r*), 7.10–7.13 (m, 6H, *h*,*q*), 7.22–7.31 (m, 6H, *d*,*f*,*l*), 7.37–7.38 (m, 6H, *e*,*m*,*n*), 7.45 (d, *J* = 8.5 Hz, 2H, *k*), 7.75 (t, *J* = 8.0 Hz, 2H, *g*), 8.70 (d, *J* = 7.5 Hz, 2H, *b*), 10.3 (s, 2H, *a*). The signals are not assignable because of conformational isomerism; ^{13}C NMR (126 MHz, $CDCl_3$) δ 17.3–17.4 (2C), 99.4–99.5 (2C), 103.0 (2C), 108.2 (2C), 108.7 (2C), 112.5 (1C), 114.7 (2C), 116.5 (2C), 117.6 (2C), 120.8 (2C), 121.9 (2C), 122.8 (2C), 123.8 (2C), 125.5 (4C), 128.5 (2C), 128.9 (4C), 129.8 (2C), 132.1 (2C), 132.2 (2C), 133.2 (2C), 134.2 (2C), 135.5 (2C), 137.3–137.4 (2C), 139.9 (2C), 144.0 (2C), 146.5 (2C), 147.0 (2C), 151.1–151.2 (2C), 152.4–152.5 (1C), 157.4 (2C), 157.5 (2C), 160.2 (2C), 162.7 (2C); ^{11}B NMR (160 MHz, $CDCl_3$) δ 41.2; HRMS (MALDI) *m/z* [M]$^+$ calcd for $C_{68}H_{44}B_3N_3O_4$ 999.3634, observed 999.3647.

Details of DH-TDDFT calculation

Grimme's B2PLYP-type double-hybrid exchange-correlation functional[21,22] has the following energy form: $E_{xc}(c_x, c_c) = (1 - c_x)E_x^{\mathrm{B88}} + c_x E_x^{\mathrm{HF}} + (1 - c_c)E_c^{\mathrm{LYP}} + c_c E_c^{\mathrm{PT2}}$, where E_x^{B88} and E_c^{LYP} are the Becke88 exchange[23] and Lee–Yang–Parr correlation[24] energies, respectively; E_x^{HF} is the Hartree–Fock (HF) exchange energy; E_c^{PT2} is the second-order perturbation correlation energy; and coefficients c_x and c_x are exchange and correlation mixing parameters. We here denote B2LYP/B2PLYP variants by B2LYP/B2PLYP ($c_x = 0.40$, $c_c = 0.23$).

Transition energies and ΔE_{ST} values were estimated for optimized S_1 geometries within double-hybrid time-dependent density functional theory (DH-TDDFT), at the TDA-B2PLYP ($c_x = 0.40$, $c_c = 0.23$)/6-31G//M062X/6-31G(d) level of theory. Due to the limit of our computation resource, we omitted virtual orbitals with energies higher than 0.6 Hartree and computed two-electron integrals in the resolution-of-the-identity approximation[25–27] for CIS(D) calculations.[28] All the DH-TDDFT computations were conducted using PySCF[24–27] with in-house CIS(D) submodules.[29]

Spin-Orbit Coupling Calculations

Using the optimized geometries in the S_1 state, the spin-orbit coupling (SOC) matrix elements $\langle S_1|\hat{H}_{SOC}|T_n\rangle$ between S_1 and T_n (n = 1, 2) were obtained performing TD-DFT calculations with the M062X functional and the TZP basis set as implemented in the ADF2021 program. Taking into account the contributions from the three degenerate triplet states ($T_{n,x}$, $T_{n,y}$, and $T_{n,z}$), $\langle S_1|\hat{H}_{SOC}|T_n\rangle$ were calculated by the square root of the sum of squares of the real and imaginary parts (*Re* and *Im*) of the matrix elements (eq. S1).

$$\langle S_1|\hat{H}_{SOC}|T_n\rangle = \sum_{\alpha=x,y,z}\left[\left(Re\langle S_1|\hat{H}_{SOC}|T_{n,\alpha}\rangle\right)^2 + \left(Im\langle S_1|\hat{H}_{SOC}|T_{n,\alpha}\rangle\right)^2\right]^{\frac{1}{2}} \qquad \text{(S1)}$$

Semi-classical Marcus Equation

The k_{RISC} value from the T_1 state was estimated according to the literature.[30] Based on the semi-classical Marcus equation, the k_{RISC} value can be denoted as:

$$k_{RISC} = \frac{2\pi}{\hbar g}\frac{V^2{}_{SOC}}{\sqrt{4\pi k_B T\lambda}}\exp\left(-\frac{(\lambda + \Delta G°)^2}{4\lambda k_B T}\right)$$

where $\hbar$, g, V_{SOC}, k_B, T, λ, and $\Delta G°$ represent the Dirac's constant, degeneracy of the initial state (g = 3 for calculating RISC rate), spin–orbit coupling, Boltzmann constant, temperature, reorganization energy, and change in free energy between the energies of the respective initial triplet and final singlet states, respectively. λ is assumed to be 0.10 eV because the structural relaxation of MR-TADF emitters is generally small because of their rigid framework.

Supplementary Notes 1: Estimation of rate constant for reverse intersystem crossing. Rate constants (k_F, k_{IC}, k_{ISC}, and k_{RISC}) were determined from the measurements of quantum yields (Φ_F and Φ_{TADF}) and lifetimes (τ_F, τ_{TADF}) and of the prompt (fluorescence) and delayed (TADF) components according to Adachi's method (equations 1–2[31], 3–5[32] and 6[33]).

DOB2-DABNA-A

$\Phi = 0.919$

$\Phi_F = 0.386$

$\Phi_{TADF} = 0.533$

$\tau_F = 6.21$ ns

$\tau_{TADF} = 1.55$ μs

$k_p = 1.61 \times 10^8$ s^{-1}	$k_p = 1/\tau_F$	(1)
$k_d = 6.45 \times 10^5$ s^{-1}	$k_d = 1/\tau_{TADF}$	(2)
$k_F = 8.58 \times 10^7$ s^{-1}	$k_F = \Phi_F/\tau_F$	(3)
$k_{IC} = 0.76 \times 10^7$ s^{-1}	$\Phi = k_F/(k_F + k_{IC})$	(4)
$k_{ISC} = 6.77 \times 10^7$ s^{-1}	$\Phi_F = k_F/(k_F + k_{IC} + k_{ISC})$	(5)
$k_{RISC} = 1.12 \times 10^6$ s^{-1}	$k_{RISC} = k_p k_d/(k_p - k_{ISC})$	(6)

DOB2-DABNA-A-NP

$\Phi = 0.758$

$\Phi_F = 0.278$

$\Phi_{TADF} = 0.480$

$\tau_F = 3.41$ ns

$\tau_{TADF} = 2.39$ μs

$k_p = 2.93 \times 10^8$ s^{-1}	$k_p = 1/\tau_F$	(1)
$k_d = 4.19 \times 10^5$ s^{-1}	$k_d = 1/\tau_{TADF}$	(2)
$k_F = 8.15 \times 10^7$ s^{-1}	$k_F = \Phi_F/\tau_F$	(3)
$k_{IC} = 2.60 \times 10^7$ s^{-1}	$\Phi = k_F/(k_F + k_{IC})$	(4)
$k_{ISC} = 1.86 \times 10^8$ s^{-1}	$\Phi_F = k_F/(k_F + k_{IC} + k_{ISC})$	(5)
$k_{RISC} = 1.15 \times 10^6$ s^{-1}	$k_{RISC} = k_p k_d/(k_p - k_{ISC})$	(6)

DOB2-DABNA-B-NP

$\Phi = 0.868$

$\Phi_F = 0.773$

$\Phi_{TADF} = 0.095$

$\tau_F = 7.54$ ns

$\tau_{TADF} = 2.99$ µs

$k_p = 1.33 \times 10^8$ s^{-1}	$k_p = 1/\tau_F$	(1)
$k_d = 3.35 \times 10^5$ s^{-1}	$k_d = 1/\tau_{TADF}$	(2)
$k_F = 1.03 \times 10^8$ s^{-1}	$k_F = \Phi_F/\tau_F$	(3)
$k_{IC} = 1.56 \times 10^7$ s^{-1}	$\Phi = k_F/(k_F + k_{IC})$	(4)
$k_{ISC} = 1.44 \times 10^7$ s^{-1}	$\Phi_F = k_F/(k_F + k_{IC} + k_{ISC})$	(5)
$k_{RISC} = 3.72 \times 10^5$ s^{-1}	$k_{RISC} = k_p k_d/(k_p - k_{ISC})$	(6)

Supplementary Notes 2: Cartesian coordinates.

DOB2-DABNA-A (S_0, C_1 symmetry)

E(B3LYP/6-31G(d)) = –2876.07413571 hartree

Center Number	Atomic Number	Atomic Type	Coordinates (Angstroms) X	Y	Z
1	6	0	4.117178	-0.625216	0.323846
2	6	0	1.392910	0.121492	0.213013
3	6	0	1.093340	-3.156621	1.787747
4	6	0	2.437177	1.055805	-0.071909
5	6	0	-0.058785	-3.787299	2.278648
6	6	0	5.071443	-3.230884	1.149192
7	6	0	-0.250937	2.081998	-0.001479
8	6	0	0.850423	2.949111	-0.180057
9	6	0	-2.612681	0.435760	0.069474
10	6	0	-1.531815	2.658105	0.178532
11	6	0	-3.836180	-1.601790	-0.326184
12	6	0	-1.373441	-0.218559	-0.215036
13	6	0	-2.679387	-2.269692	-0.776464
14	6	0	-0.288350	-3.329921	-1.782537
15	6	0	3.156132	-1.552455	0.775490
16	6	0	-4.132276	-4.361333	-1.149560
17	5	0	-0.066651	0.575130	-0.001490
18	7	0	-2.642734	1.807316	0.352466
19	7	0	2.134387	2.394179	-0.353603
20	6	0	-1.485411	-1.533725	-0.719345
21	6	0	1.819761	-1.127444	0.718372
22	8	0	-0.342743	-2.084007	-1.212105
23	8	0	0.844172	-1.937003	1.212321
24	5	0	-2.737649	-3.691046	-1.299074
25	5	0	3.556629	-2.916638	1.300550
26	6	0	-1.398610	-4.199380	-1.887055
27	6	0	2.381080	-3.731259	1.893116
28	6	0	-3.833574	-0.276662	0.066321
29	6	0	-5.188721	-3.556635	-0.663680
30	6	0	-1.691933	4.048713	0.198946
31	6	0	-0.593604	4.896752	0.001060
32	6	0	0.673258	4.342711	-0.198156
33	1	0	-4.769918	0.191315	0.334198
34	1	0	-2.674076	4.474832	0.349841
35	1	0	1.526414	4.986315	-0.347409
36	6	0	-3.898907	2.411704	0.727011
37	6	0	3.207269	3.282246	-0.733927
38	6	0	-6.492350	-4.041030	-0.497676
39	6	0	-5.751030	-6.209861	-1.260756
40	6	0	-1.155468	-5.426764	-2.548249
41	6	0	0.093040	-5.774650	-3.042692
42	6	0	1.170286	-4.888446	-2.893382
43	6	0	0.983227	-3.664990	-2.269287
44	1	0	-7.257687	-3.366615	-0.126670
45	1	0	-5.963241	-7.251653	-1.484224
46	1	0	-1.979053	-6.117334	-2.692220
47	1	0	0.236349	-6.727385	-3.544851
48	1	0	2.153847	-5.153569	-3.272046
49	1	0	1.794518	-2.954598	-2.147755

50	6	0	5.901984	-2.195400	0.661689
51	6	0	2.443138	-4.977932	2.559993
52	6	0	1.316533	-5.615327	3.058708
53	6	0	0.056637	-5.016847	2.908096
54	6	0	7.283746	-2.351289	0.494171
55	6	0	7.088042	-4.634735	1.258155
56	1	0	7.863531	-1.512469	0.122084
57	1	0	7.545435	-5.594604	1.481181
58	1	0	3.409675	-5.447699	2.705145
59	1	0	1.408624	-6.572056	3.565252
60	1	0	-0.833046	-5.510384	3.290132
61	1	0	-1.018043	-3.294797	2.156606
62	6	0	5.721135	-4.455961	1.425490
63	1	0	5.127820	-5.297929	1.764389
64	6	0	3.794276	0.659563	-0.069386
65	1	0	4.589646	1.339850	-0.337786
66	8	0	-5.045706	-2.235467	-0.308762
67	8	0	5.444046	-0.947907	0.306649
68	6	0	-6.770840	-5.366171	-0.800284
69	1	0	-7.780457	-5.746944	-0.670020
70	6	0	-4.467315	-5.706849	-1.426380
71	1	0	-3.688294	-6.381002	-1.764565
72	6	0	7.873793	-3.570146	0.796529
73	1	0	8.945269	-3.696340	0.665011
74	6	0	-5.996775	3.453509	0.100410
75	6	0	-5.416427	3.160274	2.462598
76	6	0	4.496070	4.366813	-2.476809
77	6	0	4.999262	4.794554	-0.117532
78	6	0	-4.785261	2.855601	-0.258559
79	1	0	-4.519670	2.730471	-1.304658
80	6	0	-6.290549	3.600075	1.463817
81	1	0	-7.227727	4.071977	1.753441
82	6	0	-4.208572	2.562397	2.076573
83	1	0	-3.500450	2.214647	2.823932
84	6	0	5.244418	5.003942	-1.482263
85	1	0	6.041080	5.684905	-1.776386
86	6	0	3.466792	3.499516	-2.085018
87	1	0	2.858841	2.991674	-2.828934
88	6	0	3.966308	3.926231	0.247336
89	1	0	3.742341	3.743552	1.294704
90	6	0	-0.820989	6.421872	0.012249
91	6	0	-1.827614	6.800998	-1.100332
92	6	0	-1.393078	6.844829	1.386389
93	6	0	0.476302	7.216864	-0.229614
94	1	0	-1.444573	6.516879	-2.087193
95	1	0	-2.794701	6.307010	-0.962484
96	1	0	-2.002335	7.884081	-1.102278
97	1	0	-0.694485	6.596089	2.193525
98	1	0	-1.567667	7.927716	1.409362
99	1	0	-2.343986	6.347787	1.603255
100	1	0	0.257374	8.290671	-0.214607
101	1	0	1.225816	7.021748	0.545516
102	1	0	0.922854	6.983787	-1.202722
103	6	0	4.792777	4.590468	-3.941724
104	1	0	5.440801	5.459894	-4.090351
105	1	0	5.299863	3.721386	-4.380573
106	1	0	3.873725	4.750522	-4.516795
107	6	0	5.846776	5.476220	0.931863
108	1	0	6.789758	4.936673	1.090894
109	1	0	6.105446	6.498744	0.635642

110	1	0	5.330641	5.522228	1.896028
111	6	0	-6.977907	3.912215	-0.953726
112	1	0	-7.762386	3.161631	-1.117560
113	1	0	-7.476763	4.842071	-0.659239
114	1	0	-6.483004	4.082012	-1.915170
115	6	0	-5.763602	3.308937	3.925951
116	1	0	-6.610021	3.987867	4.069199
117	1	0	-6.036314	2.342239	4.368743
118	1	0	-4.915911	3.698189	4.501276

DOB2-DABNA-A-NP (S_0, C_1 symmetry)

E(B3LYP/6-31G(d)) = –3078.995695 hartree

Center Number	Atomic Number	Atomic Type	Coordinates (Angstroms) X	Y	Z
1	6	0	1.742547	-4.001275	-0.359600
2	6	0	0.665796	-1.391185	-0.224691
3	6	0	3.882137	-0.674953	-1.792444
4	6	0	-0.131343	-2.544497	0.051073
5	6	0	4.367012	0.551038	-2.269647
6	6	0	4.447226	-4.617913	-1.185997
7	6	0	-1.481092	0.000105	0.000114
8	6	0	-2.207662	-1.203692	0.175581
9	6	0	-0.131048	2.544557	-0.050940
10	6	0	-2.207554	1.203996	-0.175374
11	6	0	1.743018	4.001143	0.359573
12	6	0	0.665984	1.391163	0.224770
13	6	0	2.543857	2.929090	0.801502
14	6	0	3.882347	0.674631	1.792352
15	6	0	2.543462	-2.929302	-0.801591
16	6	0	4.447803	4.617519	1.185807
17	5	0	0.035041	0.000020	0.000093
18	7	0	-1.497270	2.409407	-0.339943
19	7	0	-1.497537	-2.409193	0.340118
20	6	0	1.957343	1.655827	0.733541
21	6	0	1.957081	-1.655984	-0.733583
22	8	0	2.640292	0.583585	1.218143
23	8	0	2.640099	-0.583802	-1.218219
24	5	0	3.947731	3.152919	1.327618
25	5	0	3.947290	-3.153263	-1.327777
26	6	0	4.611236	1.880739	1.908833
27	6	0	4.610897	-1.881133	-1.908986
28	6	0	0.427458	3.842452	-0.035308
29	6	0	3.523200	5.574066	0.706631
30	6	0	-3.607697	1.205251	-0.183980
31	6	0	-4.297756	0.000230	0.000024
32	6	0	-3.607826	-1.204833	0.184099
33	1	0	-0.148282	4.718409	-0.297531
34	1	0	-4.169690	2.120589	-0.302852
35	1	0	-4.169877	-2.120144	0.302914
36	6	0	-2.244949	3.584101	-0.716831
37	6	0	-2.245323	-3.583722	0.717298
38	6	0	3.850361	6.926309	0.545805
39	6	0	6.093835	6.441107	1.299893
40	6	0	5.856851	1.782192	2.573331
41	6	0	6.351375	0.580561	3.058858

42	6	0	5.602538	-0.594654	2.896751
43	6	0	4.367111	-0.551394	2.269579
44	1	0	3.089895	7.608803	0.179657
45	1	0	7.104238	6.773359	1.521216
46	1	0	6.442136	2.682057	2.727684
47	1	0	7.312723	0.549093	3.564026
48	1	0	5.983617	-1.542218	3.268138
49	1	0	3.760132	-1.441360	2.138343
50	6	0	3.522555	-5.574371	-0.706776
51	6	0	5.856503	-1.782692	-2.573520
52	6	0	6.351135	-0.581096	-3.059023
53	6	0	5.602423	0.594190	-2.896856
54	6	0	3.849597	-6.926646	-0.545963
55	6	0	6.093082	-6.441656	-1.300152
56	1	0	3.089084	-7.609067	-0.179779
57	1	0	7.103444	-6.774004	-1.521519
58	1	0	6.441693	-2.682611	-2.727918
59	1	0	7.312472	-0.549710	-3.564219
60	1	0	5.983590	1.541727	-3.268222
61	1	0	3.760128	1.441062	-2.138364
62	6	0	5.744976	-5.107127	-1.461374
63	1	0	6.506705	-4.410708	-1.793836
64	6	0	0.427039	-3.842452	0.035392
65	1	0	-0.148766	-4.718352	0.297661
66	8	0	2.227248	5.277553	0.352826
67	8	0	2.226646	-5.277738	-0.352919
68	6	0	5.134696	7.356795	0.846423
69	1	0	5.394050	8.404635	0.719865
70	6	0	5.745609	5.106611	1.461130
71	1	0	6.507286	4.410120	1.793561
72	6	0	5.133877	-7.357253	-0.846640
73	1	0	5.393138	-8.405117	-0.720091
74	6	0	-3.512018	5.552107	-0.115381
75	1	0	-3.934119	6.200790	0.647077
76	6	0	-3.147668	5.012009	-2.443822
77	1	0	-3.285349	5.239806	-3.497121
78	6	0	-3.147964	-5.011249	2.444643
79	1	0	-3.285493	-5.238885	3.497997
80	6	0	-3.512795	-5.551624	0.116342
81	1	0	-3.935113	-6.200353	-0.645957
82	6	0	-6.433588	-0.811373	0.922938
83	6	0	-6.433465	0.811803	-0.923221
84	6	0	-8.271815	-2.315610	1.418750
85	6	0	-6.734248	1.688415	-3.165459
86	1	0	-9.143423	-2.869497	1.079610
87	1	0	-6.407539	1.738156	-4.200984
88	7	0	-5.717114	0.000283	-0.000015
89	6	0	-7.861493	2.401402	-2.751568
90	1	0	-8.413612	3.014758	-3.458028
91	6	0	-7.561702	1.536805	-0.507774
92	1	0	-7.876975	1.483154	0.529426
93	6	0	-6.028848	-0.892379	2.265079
94	1	0	-5.161876	-0.328477	2.594083
95	6	0	-7.861855	-2.401362	2.750792
96	1	0	-8.413964	-3.014867	3.457129
97	6	0	-2.789796	4.419525	0.262680
98	1	0	-2.640507	4.177292	1.310633
99	6	0	-3.691622	5.849497	-1.468176
100	1	0	-4.254887	6.731257	-1.760803
101	6	0	-2.424151	3.878506	-2.070073

102	1	0	-1.993898	3.219117	-2.817844
103	6	0	-8.271463	2.316109	-1.419453
104	1	0	-9.143010	2.870284	-1.080571
105	6	0	-6.028733	0.892340	-2.265326
106	1	0	-5.161847	0.328220	-2.594215
107	6	0	-7.561887	-1.536081	0.507321
108	1	0	-7.877225	-1.482115	-0.529861
109	6	0	-6.734477	-1.688622	3.164894
110	1	0	-6.407844	-1.738762	4.200439
111	6	0	-3.692211	-5.848797	1.469206
112	1	0	-4.255566	-6.730424	1.762058
113	6	0	-2.424346	-3.877907	2.070614
114	1	0	-1.993867	-3.218482	2.818224
115	6	0	-2.790470	-4.419197	-0.262000
116	1	0	-2.641328	-4.177131	-1.310012

DOB2-DABNA-B-NP (S_0, C_1 symmetry)

E(B3LYP/6-31G(d)) = -3157.63931613 hartree

Center Number	Atomic Number	Atomic Type	Coordinates (Angstroms) X	Y	Z
1	7	0	1.762782	-2.390926	-0.495169
2	7	0	5.968284	0.000013	-0.000001
3	6	0	2.458777	1.193994	0.245698
4	6	0	-1.562053	-3.972655	-0.222990
5	6	0	-0.445647	1.396868	0.031400
6	6	0	4.548567	0.000014	-0.000013
7	6	0	-4.244676	4.720895	-0.244671
8	6	0	2.458785	-1.193975	-0.245734
9	6	0	-4.244640	-4.720926	0.244681
10	6	0	-5.555907	5.197806	-0.415604
11	6	0	-3.289219	-5.638443	-0.227662
12	6	0	-5.042951	2.445045	-1.149187
13	6	0	-2.410428	-2.921676	0.212630
14	6	0	-0.445636	-1.396873	-0.031419
15	6	0	-5.555867	-5.197846	0.415620
16	6	0	-6.304788	-3.077440	1.251508
17	6	0	0.377170	-2.527272	-0.338826
18	6	0	-3.289260	5.638417	0.227674
19	6	0	-6.304815	3.077399	-1.251501
20	6	0	-5.042929	-2.445077	1.149187
21	6	0	6.680711	-1.068276	0.611038
22	6	0	7.806320	-1.626944	-0.014536
23	6	0	6.271256	-1.574577	1.855371
24	1	0	8.124954	-1.243913	-0.978809
25	1	0	5.410225	-1.136230	2.350070
26	6	0	8.091586	-3.173608	1.829522
27	1	0	8.637160	-3.986584	2.300139
28	6	0	6.680725	1.068299	-0.611030
29	6	0	7.806323	1.626967	0.014566
30	6	0	6.271300	1.574595	-1.855375
31	1	0	8.124936	1.243939	0.978847
32	1	0	5.410280	1.136246	-2.350092
33	6	0	8.091635	3.173621	-1.829495
34	1	0	8.637222	3.986593	-2.300104
35	6	0	2.526978	-3.556620	-0.879423

36	6	0	2.740778	-3.792459	-2.238822
37	6	0	3.034902	-4.429815	0.099502
38	6	0	3.978037	-5.786413	-1.697240
39	6	0	2.526943	3.556640	0.879407
40	6	0	2.740796	3.792438	2.238805
41	6	0	3.034785	4.429894	-0.099508
42	6	0	3.977959	5.786456	1.697239
43	6	0	1.727876	0.000007	-0.000018
44	6	0	0.377150	2.527271	0.338813
45	6	0	-1.562083	3.972641	0.222988
46	6	0	-2.410452	2.921657	-0.212634
47	7	0	1.762764	2.390938	0.495145
48	5	0	0.213244	0.000001	-0.000015
49	5	0	-3.875648	-3.273883	0.552412
50	5	0	-3.875675	3.273856	-0.552410
51	6	0	-0.206441	-3.796816	-0.476157
52	1	0	0.372671	-4.664183	-0.763444
53	6	0	-1.791594	-1.659357	0.263837
54	1	0	-2.399219	-0.818061	0.568603
55	6	0	-7.436795	-2.426845	1.759721
56	1	0	-8.375726	-2.969541	1.804939
57	6	0	-6.094333	-0.453685	2.138023
58	1	0	-6.006086	0.570681	2.487752
59	6	0	-4.986716	-1.113227	1.625513
60	1	0	-4.038654	-0.587621	1.605632
61	6	0	-5.915385	-6.515394	0.128199
62	1	0	-6.939923	-6.840824	0.272643
63	6	0	-3.607053	-6.963733	-0.519443
64	1	0	-2.838359	-7.638339	-0.880639
65	6	0	3.859357	-1.188381	-0.268849
66	1	0	4.422024	-2.084000	-0.491581
67	6	0	3.859350	1.188406	0.268820
68	1	0	4.422013	2.084024	0.491563
69	6	0	-1.791609	1.659344	-0.263849
70	1	0	-2.399228	0.818045	-0.568619
71	6	0	-0.206468	3.796811	0.476151
72	1	0	0.372639	4.664180	0.763438
73	6	0	-3.607103	6.963703	0.519461
74	1	0	-2.838413	7.638313	0.880658
75	6	0	-5.915434	6.515351	-0.128176
76	1	0	-6.939975	6.840774	-0.272616
77	6	0	-7.436818	2.426799	-1.759716
78	1	0	-8.375754	2.969488	-1.804929
79	6	0	-6.094342	0.453650	-2.138032
80	1	0	-6.006089	-0.570713	-2.487766
81	6	0	-4.986729	1.113198	-1.625520
82	1	0	-4.038664	0.587599	-1.605645
83	1	0	4.545588	6.660257	2.005425
84	1	0	4.545683	-6.660206	-2.005420
85	1	0	2.330206	3.094667	2.962744
86	1	0	2.330130	-3.094730	-2.962768
87	8	0	-1.987678	5.264109	0.414573
88	8	0	-6.545806	4.377112	-0.871678
89	8	0	-6.545771	-4.377156	0.871692
90	8	0	-1.987640	-5.264127	-0.414567
91	6	0	-4.927106	7.383279	0.337089
92	1	0	-5.190433	8.413126	0.562700
93	6	0	-7.328824	1.115633	-2.197879
94	1	0	-8.203892	0.607042	-2.593681
95	6	0	-4.927052	-7.383318	-0.337064

96	1	0	-5.190373	-8.413168	-0.562671
97	6	0	6.967915	2.624260	-2.450984
98	1	0	6.638768	3.002457	-3.415442
99	6	0	8.507420	2.664568	-0.597444
100	1	0	9.376478	3.085888	-0.098810
101	6	0	6.967854	-2.624248	2.450990
102	1	0	6.638684	-3.002450	3.415438
103	6	0	8.507401	-2.664550	0.597483
104	1	0	9.376468	-3.085870	0.098866
105	6	0	3.761176	-5.544068	-0.340288
106	1	0	4.163204	-6.231630	0.399672
107	6	0	3.466996	-4.908536	-2.653034
108	1	0	3.629881	-5.088813	-3.711739
109	6	0	-7.328810	-1.115676	2.197877
110	1	0	-8.203881	-0.607089	2.593678
111	6	0	3.761039	5.544156	0.340288
112	1	0	4.163002	6.231764	-0.399664
113	6	0	3.466994	4.908525	2.653024
114	1	0	3.629923	5.088767	3.711729
115	6	0	2.810629	-4.176684	1.570052
116	1	0	1.742676	-4.112285	1.806904
117	1	0	3.245004	-4.980352	2.171766
118	1	0	3.270429	-3.232204	1.883386
119	6	0	2.810427	4.176825	-1.570057
120	1	0	1.742460	4.112437	-1.806849
121	1	0	3.244766	4.980519	-2.171761
122	1	0	3.270208	3.232360	-1.883457

DABNA-1 (S_1, C_2 symmetry)

E(M062X/6-31G(d)) = -1289.73568613 hartree

Center Number	Atomic Number	Atomic Type	Coordinates (Angstroms) X	Y	Z
1	6	0	0.000000	0.000000	3.017638
2	6	0	-0.013769	1.206086	2.325311
3	6	0	-0.003834	1.202901	0.906935
4	6	0	0.000000	0.000000	0.176285
5	6	0	0.003834	-1.202901	0.906935
6	6	0	0.013769	-1.206086	2.325311
7	6	0	0.149701	2.546023	-1.177379
8	6	0	-0.149701	-2.546023	-1.177379
9	1	0	0.000000	0.000000	4.101642
10	1	0	-0.030439	2.138319	2.875605
11	1	0	0.030439	-2.138319	2.875605
12	6	0	-0.206443	-1.376945	-2.002298
13	6	0	-0.466578	-4.001620	-3.090947
14	1	0	-0.540929	-5.003134	-3.501592
15	6	0	-0.604882	-2.882291	-3.908916
16	1	0	-0.805785	-3.002903	-4.969559
17	6	0	0.206443	1.376945	-2.002298
18	6	0	0.604882	2.882291	-3.908916
19	1	0	0.805785	3.002903	-4.969559
20	6	0	0.466578	4.001620	-3.090947
21	1	0	0.540929	5.003134	-3.501592
22	6	0	-0.251467	-3.833932	-1.723975
23	1	0	-0.173267	-4.709559	-1.092149

24	6	0	0.251467	3.833932	-1.723975
25	1	0	0.173267	4.709559	-1.092149
26	5	0	0.000000	0.000000	-1.346924
27	6	0	-0.482331	-1.607392	-3.367407
28	1	0	-0.627850	-0.751033	-4.015609
29	6	0	0.482331	1.607392	-3.367407
30	1	0	0.627850	0.751033	-4.015609
31	7	0	0.000000	2.412662	0.219859
32	7	0	0.000000	-2.412662	0.219859
33	6	0	-0.066876	3.619036	0.997091
34	6	0	-1.308363	4.169318	1.300341
35	6	0	1.107913	4.221984	1.439613
36	6	0	-1.373869	5.333836	2.061459
37	1	0	-2.206198	3.679294	0.936484
38	6	0	1.035977	5.385437	2.199956
39	1	0	2.061808	3.772296	1.180757
40	6	0	-0.203833	5.941161	2.511319
41	1	0	-2.339887	5.766132	2.301969
42	1	0	1.948488	5.858181	2.548916
43	1	0	-0.257435	6.848640	3.104277
44	6	0	0.066876	-3.619036	0.997091
45	6	0	1.308363	-4.169318	1.300341
46	6	0	-1.107913	-4.221984	1.439613
47	6	0	1.373869	-5.333836	2.061459
48	1	0	2.206198	-3.679294	0.936484
49	6	0	-1.035977	-5.385437	2.199956
50	1	0	-2.061808	-3.772296	1.180757
51	6	0	0.203833	-5.941161	2.511319
52	1	0	2.339887	-5.766132	2.301969
53	1	0	-1.948488	-5.858181	2.548916
54	1	0	0.257435	-6.848640	3.104277

----

model-A (S_1, C_2 symmetry)

E(M062X/6-31G(d)) = -2560.56406600 hartree

Center Number	Atomic Number	Atomic Type	Coordinates (Angstroms) X	Y	Z
1	6	0	0.000000	3.982093	-0.241516
2	6	0	0.134915	1.392115	0.868283
3	6	0	1.452496	0.700405	-2.433353
4	6	0	-0.217786	2.532574	1.660483
5	6	0	1.824364	-0.531253	-2.980289
6	6	0	0.839647	4.647208	-2.921194
7	6	0	0.000000	0.000000	3.011471
8	6	0	-0.209748	1.191692	3.740108
9	6	0	0.217786	-2.532574	1.660483
10	6	0	0.209748	-1.191692	3.740108
11	6	0	0.000000	-3.982093	-0.241516
12	6	0	-0.134915	-1.392115	0.868283
13	6	0	-0.508416	-2.934691	-1.037218
14	6	0	-1.452496	-0.700405	-2.433353
15	6	0	0.508416	2.934691	-1.037218
16	6	0	-0.839647	-4.647208	-2.921194
17	5	0	0.000000	0.000000	1.507262
18	7	0	0.428295	-2.378460	3.026219
19	7	0	-0.428295	2.378460	3.026219

20	6	0	-0.597199	-1.677752	-0.430063
21	6	0	0.597199	1.677752	-0.430063
22	8	0	-1.175762	-0.641942	-1.097411
23	8	0	1.175762	0.641942	-1.097411
24	5	0	-0.930609	-3.167974	-2.473338
25	5	0	0.930609	3.167974	-2.473338
26	6	0	-1.355413	-1.875355	-3.209543
27	6	0	1.355413	1.875355	-3.209543
28	6	0	0.342131	-3.816888	1.090017
29	6	0	-0.283020	-5.565200	-2.009746
30	6	0	0.225128	-1.192135	5.146088
31	6	0	-0.225128	1.192135	5.146088
32	1	0	0.664576	-4.677741	1.658509
33	1	0	0.412054	-2.103379	5.700711
34	1	0	-0.412054	2.103379	5.700711
35	6	0	0.791438	-3.544451	3.787094
36	6	0	-0.791438	3.544451	3.787094
37	6	0	-0.121824	-6.919976	-2.307551
38	6	0	-1.133469	-6.531423	-4.461503
39	6	0	-1.598905	-1.724248	-4.589894
40	6	0	-1.940135	-0.507198	-5.155524
41	6	0	-2.059912	0.624313	-4.340643
42	6	0	-1.824364	0.531253	-2.980289
43	1	0	0.323365	-7.568507	-1.560549
44	1	0	-1.482625	-6.909453	-5.417012
45	1	0	-1.484428	-2.586570	-5.238063
46	1	0	-2.105730	-0.427936	-6.225133
47	1	0	-2.319921	1.585444	-4.773982
48	1	0	-1.874499	1.396966	-2.326502
49	6	0	0.283020	5.565200	-2.009746
50	6	0	1.598905	1.724248	-4.589894
51	6	0	1.940135	0.507198	-5.155524
52	6	0	2.059912	-0.624313	-4.340643
53	6	0	0.121824	6.919976	-2.307551
54	6	0	1.133469	6.531423	-4.461503
55	1	0	-0.323365	7.568507	-1.560549
56	1	0	1.482625	6.909453	-5.417012
57	1	0	1.484428	2.586570	-5.238063
58	1	0	2.105730	0.427936	-6.225133
59	1	0	2.319921	-1.585444	-4.773982
60	1	0	1.874499	-1.396966	-2.326502
61	6	0	1.274200	5.190021	-4.148831
62	1	0	1.756262	4.534577	-4.866086
63	6	0	-0.342131	3.816888	1.090017
64	1	0	-0.664576	4.677741	1.658509
65	8	0	0.144521	-5.227420	-0.749480
66	8	0	-0.144521	5.227420	-0.749480
67	6	0	-0.542911	-7.399314	-3.536248
68	1	0	-0.423296	-8.452248	-3.772165
69	6	0	-1.274200	-5.190021	-4.148831
70	1	0	-1.756262	-4.534577	-4.866086
71	6	0	0.542911	7.399314	-3.536248
72	1	0	0.423296	8.452248	-3.772165
73	6	0	-0.201061	-4.402004	4.253560
74	1	0	-1.240099	-4.183626	4.026110
75	6	0	1.502931	-5.778402	5.266569
76	1	0	1.781449	-6.653528	5.844853
77	6	0	2.134342	-3.791019	4.051540
78	1	0	2.884006	-3.103471	3.672435
79	6	0	-1.502931	5.778402	5.266569

80	1	0	-1.781449	6.653528	5.844853
81	6	0	-2.134342	3.791019	4.051540
82	1	0	-2.884006	3.103471	3.672435
83	6	0	0.201061	4.402004	4.253560
84	1	0	1.240099	4.183626	4.026110
85	6	0	0.000000	0.000000	5.833235
86	1	0	0.000000	0.000000	6.917299
87	6	0	0.159810	-5.522389	4.995993
88	1	0	-0.608418	-6.195702	5.362182
89	6	0	2.488215	-4.914248	4.794764
90	1	0	3.534271	-5.113519	5.003757
91	6	0	-0.159810	5.522389	4.995993
92	1	0	0.608418	6.195702	5.362182
93	6	0	-2.488215	4.914248	4.794764
94	1	0	-3.534271	5.113519	5.003757

---

model-B (S_1, C_2 symmetry)

E(M062X/6-31G(d)) = -2560.56610457 hartree

Center Number	Atomic Number	Atomic Type	Coordinates (Angstroms) X	Y	Z
1	7	0	-0.119109	2.418873	3.452947
2	6	0	0.057214	-1.209741	4.153769
3	6	0	0.109736	3.962539	0.116045
4	6	0	-0.121107	-1.378252	1.249511
5	6	0	-0.296570	-4.580342	-2.622699
6	6	0	-0.057214	1.209741	4.153769
7	6	0	0.296570	4.580342	-2.622699
8	6	0	-0.290190	-4.980851	-3.965445
9	6	0	0.029868	5.560586	-1.657885
10	6	0	-0.962406	-2.223653	-3.417856
11	6	0	0.376506	2.859942	-0.733411
12	6	0	0.121107	1.378252	1.249511
13	6	0	0.290190	4.980851	-3.965445
14	6	0	0.869531	2.781279	-4.710389
15	6	0	-0.016280	2.545577	2.070119
16	6	0	-0.029868	-5.560586	-1.657885
17	6	0	-0.869531	-2.781279	-4.710389
18	6	0	0.962406	2.223653	-3.417856
19	6	0	0.000000	0.000000	3.424151
20	6	0	0.016280	-2.545577	2.070119
21	6	0	-0.109736	-3.962539	0.116045
22	6	0	-0.376506	-2.859942	-0.733411
23	7	0	0.119109	-2.418873	3.452947
24	5	0	0.000000	0.000000	1.920449
25	5	0	0.573345	3.139230	-2.233023
26	5	0	-0.573345	-3.139230	-2.233023
27	6	0	-0.051367	3.826123	1.491307
28	1	0	-0.208879	4.722428	2.076141
29	6	0	0.363256	1.600356	-0.110186
30	1	0	0.551716	0.737890	-0.731635
31	6	0	1.140635	2.041698	-5.865942
32	1	0	1.034197	2.526285	-6.830721
33	6	0	1.707854	0.145640	-4.484524
34	1	0	2.032643	-0.885875	-4.392282
35	6	0	1.411698	0.886881	-3.353674

36	1	0	1.538442	0.419859	-2.382161
37	6	0	0.040565	6.296815	-4.345063
38	1	0	0.043716	6.565045	-5.395231
39	6	0	-0.212932	6.886706	-1.995197
40	1	0	-0.410197	7.614920	-1.216945
41	6	0	-0.061993	1.210826	5.562531
42	1	0	-0.113065	2.138675	6.118377
43	6	0	0.061993	-1.210826	5.562531
44	1	0	0.113065	-2.138675	6.118377
45	6	0	-0.363256	-1.600356	-0.110186
46	1	0	-0.551716	-0.737890	-0.731635
47	6	0	0.051367	-3.826123	1.491307
48	1	0	0.208879	-4.722428	2.076141
49	6	0	0.212932	-6.886706	-1.995197
50	1	0	0.410197	-7.614920	-1.216945
51	6	0	-0.040565	-6.296815	-4.345063
52	1	0	-0.043716	-6.565045	-5.395231
53	6	0	-1.140635	-2.041698	-5.865942
54	1	0	-1.034197	-2.526285	-6.830721
55	6	0	-1.707854	-0.145640	-4.484524
56	1	0	-2.032643	0.885875	-4.392282
57	6	0	-1.411698	-0.886881	-3.353674
58	1	0	-1.538442	-0.419859	-2.382161
59	8	0	0.000000	-5.245075	-0.329635
60	8	0	-0.517423	-4.081980	-4.960020
61	8	0	0.517423	4.081980	-4.960020
62	8	0	0.000000	5.245075	-0.329635
63	6	0	0.203967	-7.235851	-3.345662
64	1	0	0.396535	-8.266953	-3.625213
65	6	0	-1.555474	-0.725995	-5.748133
66	1	0	-1.763374	-0.146050	-6.642384
67	6	0	-0.203967	7.235851	-3.345662
68	1	0	-0.396535	8.266953	-3.625213
69	6	0	1.555474	0.725995	-5.748133
70	1	0	1.763374	0.146050	-6.642384
71	6	0	0.000000	0.000000	6.248293
72	1	0	0.000000	0.000000	7.332468
73	6	0	0.250802	-3.625773	4.226231
74	6	0	-0.890780	-4.316672	4.621351
75	6	0	1.519734	-4.081921	4.567895
76	6	0	-0.757054	-5.480948	5.372360
77	1	0	-1.866518	-3.937227	4.333630
78	6	0	1.645927	-5.247224	5.319439
79	1	0	2.390504	-3.521920	4.241083
80	6	0	0.509492	-5.945599	5.721484
81	1	0	-1.642764	-6.025348	5.683388
82	1	0	2.632696	-5.609460	5.588953
83	1	0	0.610740	-6.854258	6.306184
84	6	0	-0.250802	3.625773	4.226231
85	6	0	-1.519734	4.081921	4.567895
86	6	0	0.890780	4.316672	4.621351
87	6	0	-1.645927	5.247224	5.319439
88	1	0	-2.390504	3.521920	4.241083
89	6	0	0.757054	5.480948	5.372360
90	1	0	1.866518	3.937227	4.333630
91	6	0	-0.509492	5.945599	5.721484
92	1	0	-2.632696	5.609460	5.588953
93	1	0	1.642764	6.025348	5.683388
94	1	0	-0.610740	6.854258	6.306184

Supplementary References

1. Hatakeyama, T. et al. Ultrapure blue thermally activated delayed fluorescence molecules: efficient HOMO-LUMO separation by the multiple resonance effect. *Adv. Mater.* **28**, 2777–2781 (2016).
2. Park, I. S., Yang, M., Shibata, H., Amanokura, N. & Yasuda, T. Achieving ultimate narrowband and ultrapure blue organic light-emitting diodes based on polycyclo-heteraborin multi-resonance delayed-fluorescence emitters. *Adv. Mater.* **34**, 2107951 (2022).
3. Park, I. S., Min, H. & Yasuda, T. Ultrafast triplet–singlet exciton interconversion in narrowband blue organoboron emitters doped with heavy chalcogens. *Angew. Chem. Int. Ed.* **61**, e202205684 (2022).
4. Park, J. et al. Asymmetric blue multiresonance TADF emitters with a narrow emission band. *ACS Appl. Mater. Interfaces* **13**, 45798 (2021).
5. Patil, V. V. et al. Purely spin-vibronic coupling assisted triplet to singlet up-conversion for real deep blue organic light-emitting diodes with over 20% efficiency and y color coordinate of 0.05. *Adv. Sci.* **8**, 2101137 (2021).
6. Suresh, S. M. et al. A deep-blue-emitting heteroatom-doped MR-TADF nonacene for high-performance organic light-emitting diodes. *Angew. Chem. Int. Ed.* **62**, e202215522 (2023).
7. Wang, X. et al. Mesityl-functionalized multi-resonance organoboron delayed fluorescent frameworks with wide-range color tunability for narrowband OLEDs. *Angew. Chem. Int. Ed.* **61**, e202206916 (2022).
8. Cao, C. et al. Intramolecular cyclization: a convenient strategy to realize efficient BT.2020 blue multi-resonance emitter for organic light-emitting diodes. *Angew. Chem. Int. Ed.* **62**, e202215226 (2023).
9. Chan, C. Y. et al. Two boron atoms versus one: high-performance deep-blue multi-resonance thermally activated delayed fluorescence emitters. *Chem. Commun.* **58**, 9377–9380 (2022).
10. Luo, M. et al. Frontier molecular orbitals regulation enables efficient and ultraviolet to deep-blue narrowband emission. *Adv. Opt. Mater.* **11**, 2202176 (2023).
11. Suresh, S. M. et al. Judicious heteroatom doping produces high performance deep blue/near UV multiresonant thermally activated delayed fluorescence OLEDs. *Adv. Mater.* **35**, 2300997 (2023).
12. Han, J. et al. Simple Molecular Design Strategy for Multiresonance Induced TADF Emitter: Highly Efficient Deep Blue to Blue Electroluminescence with High Color Purity. *Adv. Optical Mater.* **10**, 2102092 (2022).
13. Lv, X. et al. Extending the π-skeleton of multi-resonance TADF materials towards high-efficiency narrowband deep-blue emission. *Angew. Chem. Int. Ed.* **61**, e202201588 (2022).
14. Kim, J. H., Chung, W. J., Kim, J. & Lee, J. Y. Concentration quenching–resistant multiresonance thermally activated delayed fluorescence emitters. *Materials Today Energy* **21**, 100792 (2021).

15. Naveen, K. R. et al. Deep blue diboron embedded multi-resonance thermally activated delayed fluorescence emitters for narrowband organic light emitting diodes. *Chem. Eng. J.* **432**, 134381 (2022).
16. Naveen, K. R. et al. Modular design for constructing narrowband deep-blue multiresonant thermally activated delayed fluorescent emitters for efficient organic light emitting diodes. *Chem. Eng. J.* **451**, 138498 (2023).
17. Wang, Y. et al. The selective regulation of borylation site based on one-shot electrophilic C–H borylation reaction, achieving highly efficient narrowband organic light-emitting diodes. *Chem. Eng. J.* **431**, 133221 (2022).
18. Wang, Y. et al. A periphery cladding strategy to improve the performance of narrowband emitters, achieving deep-blue OLEDs with CIEy < 0.08 and external quantum efficiency approaching 20%. *Org. Electron.* **97**, 106275 (2021).
19. Chang, Y. et al. Boron, sulfur-doped polycyclic aromatic hydrocarbon emitters with multiple-resonance-dominated lowest excited states for efficient narrowband deep-blue emission. *Chem. Eng. J.* **451**, 138545 (2023).
20. Lee, H. et al. Manipulating Spectral Width and Emission Wavelength towards Highly Efficient Blue Asymmetric Carbazole Fused Multi-Resonance Emitters. *ACS Appl. Mater. Interfaces* **14**, 36927 (2022).
21. Grimme, S. Semiempirical hybrid density functional with perturbative second-order correlation. *J. Chem. Phys.* **124**, 034108 (2006).
22. Grimme, S. & Neese, F. Double-hybrid density functional theory for excited electronic states of molecules. *J. Chem. Phys.* **127**, 154116 (2007).
23. Becke, A. D. Density-functional exchange-energy approximation with correct asymptotic behavior. *Phys. Rev. A* **38**, 3098 (1988).
24. Lee, C., Yang, W. & Parr, R. G. Development of the Colle-Salvetti correlation-energy formula into a functional of the electron density. *Phys. Rev. B* **37**, 785 (1988).
25. Feyereisen, M., Fitzgerald, G. & Komornicki, A. Use of approximate integrals in ab initio theory. An application in MP2 energy calculations. *Chem. Phys. Lett.* **208**, 359 (1993).
26. Vahtras, O., Almlöf, J. & Feyereisen, M. W. Integral approximations for LCAO-SCF calculations *Chem. Phys. Lett.* **213**, 514 (1993).
27. Weigend, F. & Häser, M. RI-MP2: first derivatives and global consistency. *Theor. Chem. Acc.* **97**, 331 (1997).
28. Head-Gordon, M., Rico, R. J., Oumi M. & Lee, T. J. A doubles correction to electronic excited states from configuration interaction in the space of single substitutions. *Chem. Phys. Lett.* **219**, 21 (1994).
29. Kondo, M. Singlet-triplet energy gap of multiresonant molecular systems: A double hybrid time-

dependent density functional theory study. *Chem. Phys. Lett.* **804**, 139895 (2022).

30. Cho, E., Liu, L., Coropceanu, V. & Brédas. J.-L. Impact of secondary donor units on the excited-state properties and thermally activated delayed fluorescence (TADF) efficiency of pentacarbazole-benzonitrile emitters. *J. Chem. Phys.* **153**, 144708 (2020).
31. Masui, K., Nakanotani, H., Adachi, C. Analysis of exciton annihilation in high-efficiency sky-blue organic light-emitting diodes with thermally activated delayed fluorescence. *Org. Electron.* **14**, 2721 (2013).
32. Zhang, Q. et al. Anthraquinone-Based Intramolecular Charge-Transfer Compounds: Computational Molecular Design, Thermally Activated Delayed Fluorescence, and Highly Efficient Red Electroluminescence. *J. Am. Chem. Soc.* **136**, 18070 (2014).
33. Kaji, H. et al. Purely organic electroluminescent material realizing 100% conversion from electricity to light. *Nat. Commun.* **6**, 8476 (2015).
